# Multispecies transcriptomes reveal core fruit development genes

**DOI:** 10.3389/fpls.2022.954929

**Published:** 2022-11-04

**Authors:** Alex Rajewski, Dinusha C. Maheepala, Jessica Le, Amy Litt

**Affiliations:** Department of Botany and Plant Sciences, University of California Riverside, Riverside, CA, United States

**Keywords:** dry fruit, fleshy fruit, transcriptome, Solanaceae, Arabidopsis, melon, tobacco, tomato

## Abstract

During angiosperm evolution there have been repeated transitions from an ancestral dry fruit to a derived fleshy fruit, often with dramatic ecological and economic consequences. Following the transition to fleshy fruits, domestication may also dramatically alter the fruit phenotype *via* artificial selection. Although the morphologies of these fruits are well documented, relatively less is known about the molecular basis of these developmental and evolutionary shifts. We generated RNA-seq libraries from pericarp tissue of desert tobacco and both cultivated and wild tomato species at common developmental time points and combined this with corresponding, publicly available data from Arabidopsis and melon. With this broadly sampled dataset consisting of dry/fleshy fruits and wild/domesticated species, we applied novel bioinformatic methods to investigate conserved and divergent patterns of gene expression during fruit development and evolution. A small set of 121 orthologous “core” fruit development genes show a common pattern of expression across all five species. These include key players in developmental patterning such as orthologs of *KNOLLE*, *PERIANTHIA*, and *ARGONAUTE7*. GO term enrichment suggests that these genes function in basic cell division processes, cell wall biosynthesis, and developmental patterning. We furthermore uncovered a number of “accessory” genes with conserved expression patterns within but not among fruit types, and whose functional enrichment highlights the conspicuous differences between these phenotypic classes. We observe striking conservation of gene expression patterns despite large evolutionary distances, and dramatic phenotypic shifts, suggesting a conserved function for a small subset of core fruit development genes.

## Introduction

Seed-bearing fruits are the hallmark feature uniting the angiosperms, and this innovation has contributed to the enormous success of the group in terms of both species richness and economic importance for humans. Indeed, 82% of daily calories eaten by humans are derived directly from angiosperm plants (FAO, 2017) and 80% of those calories are from the fruits themselves. When indirect sources are taken into account, nearly all calories eaten by humans derive from angiosperms.

From a diversity standpoint, angiosperms also represent an unparalleled evolutionary success story. Since their initial split with gymnosperms, angiosperms diversified prolifically to comprise approximately 90% of all extant land plant species and now occupy key positions in nearly every biome on the planet (Crepet and Niklas, 2009). Although the precise reasons for the evolutionary diversification and success of angiosperms are still debated (Armbruster, 2014), certainly the complex interplay between flowers and their pollinators and the ability to further use animals as a seed dispersal vectors has contributed significantly to this (Regal, 1977).

Although the molecular mechanisms underlying fruit development and evolution are not thoroughly understood, morphological changes are well documented and provide a conceptual framework to examine molecular mechanisms. Fruits can broadly be classified as either dry or fleshy. The true berry of cultivated tomato (*Solanum lycopersicum*) and the pepo of melon (*Cucumis melo*) are examples of fleshy fruits, while the capsules of desert tobacco (*Nicotiana obtusifolia*) and the silique of the model plant *Arabidopsis thaliana* (hereafter, Arabidopsis) are both dry fruits. Despite the very different appearances of these fruits, the developmental progression of each can be divided into common stages with similar processes occurring at each stage across all four species (**Table 1**) (Gillaspy et al., 1993).

**Table 1:** Developmental stages of fruit development in the study species and data sources.

Description of Developmental Stages				
	Desert Tobacco	*A. thaliana*	Tomato	Melon
Stage 1	Ovary patterning (0 DAP)	Ovary patterning (3 DAP)	Ovary patterning (1 DAP)	Ovary patterning (—)*^1^*
Stage 2	Transverse anticlinal cell division (3 DAP)	Cell division and expansion (6 DAP)	Anticlinal and periclinal cell division (3 DAP)	Cell division (10 DAP)
Stage 3	Beginning basipetal lignification (6 DAP)	Beginning basipetal lignification (9 DAP)	Cell expansion and endoreduplication (15 DAP)	Cell expansion (20 DAP)
Transition	Color change from green to brown (11 DAP)	Color change to from green to yellow (12 DAP)	Initial color change from green to red, often termed 'breaker' stage (35 DAP)	Increase in sugar content, maximum firmness (30 DAP)
Stage 4	Senescence and dehiscence (—)*^1^*	Senescence and dehiscence (—)*^1^*	Cell wall softening, increase in sugar content, and full accumulation of pigments (45 DAP)	Fruit softening, maximum sugar content (40 DAP)
Bioproject Accession	PRJNA646747 (This Study)	PRJEB25745 (Mizzotti et al, 2018)	PRJNA646747 (This Study)	PRJNA314069 (Chayut et al, 2017)

^1^Not sampled. Study species are shown in bold in the first row with stage names in the first column. Intersections of species and stage provide a brief description of developmental hallmarks and the number of days after pollination (DAP) when this stage occurs and when sampling occurred. NCBI bioproject accession numbers for each data source are provided in the final row.

Stage descriptions adapted from: Pabón-Mora and Litt, 2011; Mizzotti et al, 2018; and Zhang et al, 2016.

All fruits are derived from one or multiple ovaries. The earliest stage of fruit development (Stage 1) occurs before the ovules have been fertilised and comprises a stage of ovary patterning that is common to all species. Although specific terminology differs, the ovaries of all four species previously mentioned are divided into multiple chambers. In the cases of desert tobacco, Arabidopsis, and the wild relative of tomato (*S. pimpinellifolium*), the ovary is divided into two chambers. The fruits of wild melon species have 2-5 chambers, while both cultivated melon and cultivated tomato have a variable number of chambers (Monforte et al., 2014). Following fertilisation of the ovules, the ovary transitions to a fruit and enters into a stage of rapid cell division (Stage 2). The length of this phase differs, with both Arabidopsis and desert tobacco undergoing cell division phases of 1-3 days, while tomato and melon cell division phases can occur over 1-2 weeks (Pabón-Mora and Litt, 2011; Chayut et al., 2015; Ripoll et al., 2019). Additionally, the orientation of these cell divisions in the pericarp (outer fruit wall) varies. Pericarp cell divisions in desert tobacco are primarily anticlinal and maintain 7-8 pericarp cell layers, but pericarp divisions in tomato, and likely melon, are both anticlinal and periclinal and increase the number of cell layers dramatically (Pabón-Mora and Litt, 2011; Shin et al., 2017).

Following this burst of cell division, the fruit enters a phase of cell differentiation (Stage 3). In this stage, the fruits of each species begin to morphologically diverge from one another more drastically. Among the dry-fruited species Arabidopsis and desert tobacco, Stage 3 is characterised primarily by the deposition of lignin in the secondary cell walls of the pericarp. Because both of these fruits are dehiscent, pericarp lignification is tightly spatially controlled to allow for the formation of dehiscence zones where the mature pericarp will split open to allow seed dispersal (Ferrándiz et al., 2000; Smykal et al., 2007). In tomato and melon, Stage 3 of fruit development is characterized by pronounced pericarp cell expansion and contributes strongly to the mature fruit size. Concomitant with the increase in cell volume is also an increase in cell ploidy, with endoreduplication up to 256x (Bourdon et al., 2010). Endoreduplication has also been reported in Arabidopsis pericarp cells undergoing cell expansion and may be a more general feature of Stage 3 across fruit types (Ripoll et al., 2019).

Having reached their final size, these fruits transition to physiological maturity (Stage 4). In the case of the dry fruits presented here, Stage 4 involves senescence, drying down, and dehiscence of the pericarp along the previously patterned dehiscence zones. During dehiscence, tension created by drying of the lignified pericarp and autolysis of certain cells in the dehiscence zone allow the pericarp to split open and seeds to be dispersed. In contrast, Stage 4 in fleshy fruits generally involves accumulation of sugars, volatile and flavour compounds, pigments, and nutrients in the pericarp, along with softening of pericarp cell walls. In the climacteric fruits tomato and melon, this process coincides with a burst in production of the gaseous hormone ethylene, but non-climacteric fruits undergo similar processes in an ethylene-independent manner. Especially in tomatoes, an initial transition or “breaker” stage is also recognized between Stages 3 and 4. Breaker stage is characterised by the initial colour change in the pericarp from green to pink or red.

The early morphological similarities and the similar early developmental processes occurring across these diverse fruit types are likely related to their shared evolutionary origin. In fact across angiosperm evolution, there have been repeated shifts from an ancestral dry fruit to a derived fleshy fruit (Cox, 1948; Bremer and Eriksson, 1992; Plunkett et al., 1997; Clausing et al., 2000; Spalik et al., 2001; Knapp, 2002; Weber, 2004; Givnish et al., 2005) The conservation of morphological, developmental, and evolutionary patterns led us to hypothesise that there might also be conservation of gene function and/or gene expression patterns in fruit development across species. Although many studies characterising gene expression during fruit development have dramatically advanced our understanding within single species or between closely related species, a comparison at higher taxonomic levels could provide evidence for a set of “core” fruit development genes and shed light on the conserved pathways necessary to build a fruit.

We examined pericarp transcriptomes of two dry- and three fleshy-fruited species across developmental time. Our results draw upon 42 pericarp RNAseq libraries of three members of the nightshade family (Solanaceae) generated for this study as well as data from 30 additional publically available pericarp libraries of more distantly related dry- and fleshy-fruited species (**Table 1**). Integrating information about orthologous genes and using nested models to call differential gene expression across developmental stages, we uncovered a set of 121 genes with conserved patterns of expression among these species. These genes participate in many biological processes and may constitute a core set of genes whose expression patterns are necessary (but not sufficient) for fruit development. In addition, we found a much larger set of 1,795 genes with patterns of expression conserved within, but divergent between, dry and fleshy fruits. These genes with divergent patterns between fruit types may represent accessory genes that act to specify the developmental patterns separating these fruit types.

## Methods

### Plant materials

Seeds for *Solanum lycopersicum* ‘Ailsa Craig’ and *Solanum pimpinellifolium* (LA 2547) were provided by the UC Davis Tomato Genetics Resource Center, and those for *Nicotiana obtusifolia* (TW143) were obtained from the New York Botanical Garden. We grew all plants in a temperature-controlled greenhouse at 26°C on the campus of the University of California, Riverside.

### Developmental staging

For *Solanum* spp., we chose five developmental time points for sampling, corresponding to widely accepted stages in fruit development (Gillaspy et al., 1993): early ovary development until fruit set, initiation of cell division, initiation of cell differentiation, and ripening or maturity. For *Solanum* spp., we divided the ripening stage into a transition or “breaker” stage and true physiological maturity. The same schema was applied in the dry-fruited *N. obtusifolia*, except for physiological maturity, which is highly lignified and fully senesced. Because of the difficulty obtaining usable RNA from this stage, we did not include it for *N. obtusifolia* (**Table 1**).

To determine the timing of the early stages, we conducted serial sectioning and staining on a series of greenhouse-grown pericarps from each species. We collected fruit and ovary tissue from 0-15 days post anthesis (DPA) and trimmed them to roughly 1cm cubes as needed. We vacuum infiltrated (-0.08Mpa) these in FAA consisting of 10% formaldehyde, 50% ethanol, and 5% acetic acid in distilled water overnight and then stored them in 50% ethanol for later use. Before embedding the fixed tissue for sectioning, we first dehydrated it through an ethanol series ending with a final absolute ethanol dehydration overnight. Across two two-hour incubations at room temperature, we replaced the ethanol with 50% ethanol/50% Citrisolv (Decon Labs, King of Prussia, PA) followed by 100% Citrisolv. We then added paraffin chips, placed the samples in a 60°C oven, and replaced the solution with liquid paraffin approximately seven times over the next two days. After we could no longer smell the Citrisolv, we placed the tissue in aluminium crinkle dishes (VWR, Radnor, PA) to solidify before shaping and mounting them for sectioning the next day. We sectioned the blocks into 8-10µM thick ribbons and affixed them to microscope slides.

We stained high-quality, representative sections with Safranin O and Astra Blue. To deparaffinize the tissue slides we washed them twice for five minutes each in xylene, and followed this by rehydration through an ethanol series. We first stained in Safranin O (1% w/v in water) for 60 minutes, rinsed them twice with deionized water and then counterstained with Astra Blue (1% w/v in a 2% tartaric acid solution) for 10 minutes. We then rinsed the slides twice in water, and dehydrated them through the same ethanol series before rinsing twice with xylene. We then affixed a coverslip with permount and dried the slides at 40° overnight. We imaged the slides to count cell layers and observe cell size increases in the case of *Solanum* spp. and to observe lignification in the case of *N. obtusifolia*.

To determine the timing of stage 2 (cell division) in *N. obtusifolia* we observed fruits for a conspicuous jump in size and a shift in fruit apical shape from conical to blunted.

### RNA extraction and sequencing

For all three species, we hand-dissected pericarps on ice from developing fruits and, in the case of earlier developmental stages, pooled multiple pericarps from a single individual to obtain enough tissue for RNA isolation. Each biological replicate represents pericarps from a single plant. We snap froze dissected tissue in liquid nitrogen, ground each sample with a micropestle attached to a cordless drill, and isolated RNA with the RNeasy Plant Mini Kit (QIAGEN, Hilden, Germany) according to the manufacturer’s protocol. For *N. obtusifolia* the lysis step of this protocol was modified to use buffer RLC instead of RLT and supplemented with 2.5% (w/v) polyvinylpyrrolidone (PVP). DNA contamination was removed with an on-column RNAse-Free DNAse kit (QIAGEN, Hilden, Germany) according to the manufacturer’s protocol.

The UCR Institute for Integrative Genome Biology (IIGB) Genomics Core assessed the integrity of the isolated RNA using an Agilent 2100 Bioanalyzer. We prepared high-quality samples into Illumina RNA-sequencing libraries using the NEBNext Ultra II Directional RNA library Prep Kit for Illumina (New England BioLabs, Ipswich, MA, United States) and barcoded each library for multiplexing with the NEBNext Multiplex Oligos for Illumina (Index Primers Set 1) kit. Both protocols were undertaken according to the manufacturer’s instructions.

Libraries for *S. lycopersicum*, *S. pimpinellifolium*, and *N. obtusifolia* were sequenced at the UCR IIGB Genomics Core. All *Solanum* libraries and the stage 1-3 libraries of *N. obtusifolia* were sequenced on the Illumina NextSeq V2 with a high-output 2x75bp run. The Stage 4 libraries were sequenced as part of an Illumina NextSeq 1x75bp run. Raw sequence reads for all 42 pericarp libraries are available under NCBI BioProject PRJNA646747.

### Bioinformatic analyses

All scripts used to analyse RNA-seq data for this study are publically accessible in a GitHub repository (github.com/rajewski/SolTranscriptomes).

We downloaded the raw RNA-seq reads for the *Arabidopsis thaliana* and Cucumis melon experiments (PRJEB25745 and PRJNA314069, respectively, **Table 1**) from the Sequence Read Archive (Chayut et al., 2017; Mizzotti et al., 2018). We trimmed the demultiplexed RNA-seq data with TrimGalore (Krueger, 2012) and mapped reads using STAR v2.5.3a (Dobin et al., 2013). Because of the low continuity of the *S, pimpinellifolium* reference genome, we mapped RNA-seq reads for both *Solanum* species to the *S. lycopersicum* (SL4.0) genome assembly (Hosmani et al., 2019). For *N. obtusifolia*, we mapped the reads to version 1 of the *Nicotiana obtusifolia* reference genome assembly (Xu et al., 2017), for *Arabidopsis thaliana* data, we mapped reads to the TAIR10 assembly (Berardini et al., 2015), and for melon, we mapped read to the *Cucumis melo* cv. DHL92 genome (Garcia-Mas et al., 2012).

We used the program OrthoFinder2 (Emms and Kelly, 2019) to cluster the genes from the five species into orthologous groups based on protein sequence similarity. Within the framework of the OrthoFinder2 pipeline, we opted for gene tree estimation using multiple sequence alignments with MAFFT (Katoh and Standley, 2013) followed by IQ-Tree (Nguyen et al., 2015) instead of the default DendroBLAST algorithm (Kelly and Maini, 2013). To obtain a more tractable dataset for differential expression analyses, we eliminated orthologous groups with paralogs and filtered the results for single copy genes common to all species.

Because our experimental design contained several sequential timepoints and multiple species, pairwise comparisons with time points coded as unrelated categorical variables would fail to intuitively capture the dynamic nature of gene expression and would suffer from a severe multiple testing problem. Similarly, treating time as a linear predictor of gene expression would fail to identify transiently up-regulated genes. To avoid this problem, we opted instead to implement a natural cubic spline basis transform of the time coordinates, as outlined in the supplemental material of (Fischer et al., 2018). For differential expression testing, a gene (or orthogene) is determined to be differentially expressed if its expression profile is better fit by this spline model than by a model incorporating only noise, as determined by a likelihood ratio test. Additionally, for orthogene comparisons between fruit types, an orthogene may be differentially expressed if its expression profile is statistically significantly better fit by a model incorporating interaction between the fruit type (categorical) variable and the spline basis function coefficients than by a model with only the spline coefficients. We conducted these analyses in R using the DESeq2 and splines packages (Love et al., 2014; R Core Team, 2019). We then clustered genes determined to be differentially expressed using the DIANA algorithm of divisive clustering (Kaufman and Rousseeuw, 2005) as implemented by the R package DEGreport (Pantano, 2019). We interrogated groups of similarly expressed genes using several methods. To test for enrichment of Gene Ontology (GO) terms, we queried all protein sequences extracted from the reference genomes against the PFAM, ProSiteProfiles, TIGRFAM, and PRINTS databases (Haft et al., 2001; Attwood et al., 2012; Sigrist et al., 2013; El-Gebali et al., 2019) and aggregated all associated GO terms for each protein using a custom bash script. We then used the R package topGO (Alexa and Rahnenfuhrer, 2016) to test for enrichment of GO terms using Fisher’s Exact Test and the “weight01” algorithm against a background set of all GO terms in the genome (or in the set of orthologous genes) using a custom R script.

## Results

### Expression patterns for polyamine and isoprenoid biosynthesis are conserved between wild and cultivated tomato species

In our investigation, we began with the commonly studied cultivated tomato (*Solanum lycopersicum*) but also included its closest wild relative (*S*. *pimpinellifolium*). We reasoned that the intentional and unintentional changes during the domestication of cultivated tomato could have an impact on gene expression patterns in the fruit, whose ripening, flavour, and structure have been targets of artificial selection.

**Figure 1:**
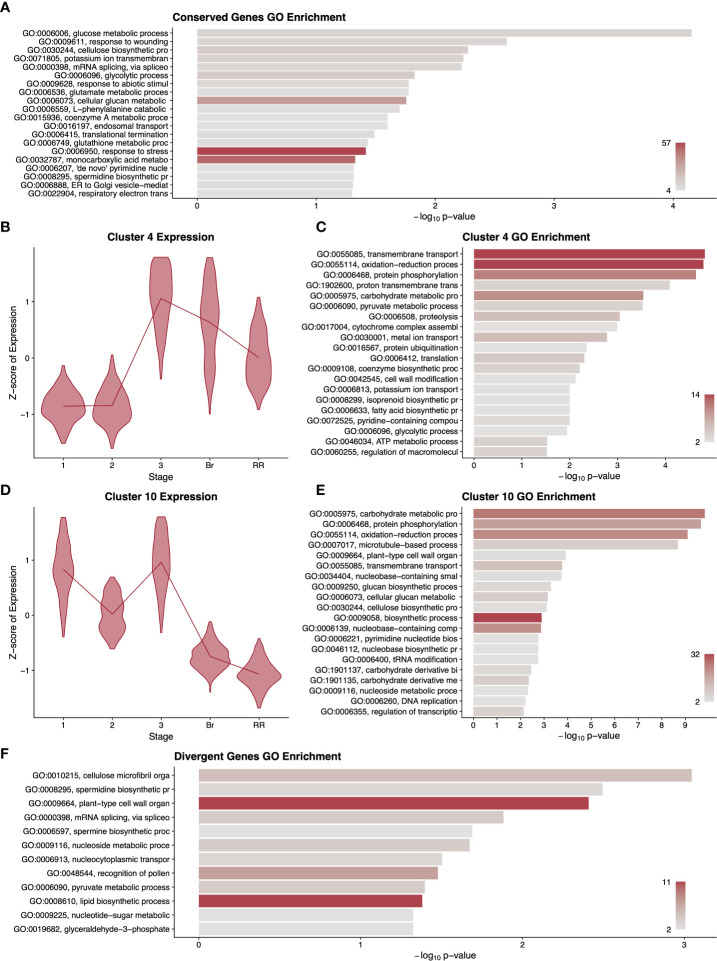
Summary of gene expression patterns conserved **(A–E)** or divergent **(F)** among cultivated and wild tomato. A gene ontology (GO) term enrichment analysis **(A)** performed on all differentially expressed genes without regard to species. Selected clusters of differentially expressed genes conserved among species are described with violin plots of normalised expression at each stage of development **(B, D)** and with GO enrichment analyses **(C, E)**, corresponding to 1415 and 1825 genes respectively. For differentially expressed genes with divergent expression between the species, we performed a GO enrichment analysis **(F)**. GO term descriptions to the left of the enrichment graphs are truncated for space and sorted by p-value. The bars are colored by the number of genes assigned to each GO term with legends in the lower right of each graph. Stages of fruit development in the axis of B and D are numbered sequentially followed by “Br” for breaker stage and “RR” for red ripe stage.

Using RNAseq data from five developmental stages from fruit of both tomato species (**Table 1**), we first asked which differentially expressed genes across fruit development showed a conserved pattern of expression between the two species. We aligned reads from both tomato species to the most recent annotation of the cultivated tomato genome. We chose to use the cultivated tomato reference genome for *S. pimpinellifolium* mapping because existing *S. pimpinellifolium* genomic resources lack the contiguity, thorough annotation, and/or data availability provided by the cultivated tomato reference genome. This also had the added benefit of simplifying ortholog determination between the two tomato species. **Supplemental File 3** shows the mapping statistics for all sequencing libraries used in this study. Libraries from both tomato species showed nearly identical percentages of mapped reads indicating a negligible bias due to reference genome choice. We then called differential expression among developmental stages with a model that was blind to species (Sander et al., 2017; Fischer et al., 2018; Hosmani et al., 2019). This model required that the expression of a gene be statistically significantly different between at least two stages. We discovered 6,165 genes (of 34,075 total) with changes in pericarp expression level with the same pattern in cultivated and wild tomato. A GO term enrichment analysis of this cohort of genes revealed that they function in diverse general biological processes including glucose metabolism, transport, and responses to damage and stress (**Figure 1A**). In addition, several lower-level GO terms were also enriched among this set of genes including spermidine biosynthetic processes, which play a role in the synthesis of polyamine compounds related to flavour and timing of fruit senescence (Nambeesan et al., 2010).

To uncover more fine-scale patterns among these differentially expressed genes, we clustered them by their expression profiles during fruit development and performed GO analyses on each of the 20 resulting clusters (**Supplementary Data Figure S1**). Several of these clusters showed informative enrichments. Cluster 4 contained genes with low and steady expression in early fruit development, peaking at the transition to Stage 3 and remaining high through the red ripe stage (**Figure 1B**). This cluster showed enrichment for isoprenoid biosynthesis (GO:0008299), fatty acid biosynthesis, and potassium ion transport (**Figure 1C**). Given the peak expression of this cluster prior to the breaker stage, it is likely that these terms relate to the accumulation of pigment and flavour compounds before and during ripening (Adams et al., 1978; Tieman et al., 2012; Li et al., 2020) (Tieman et al., 2012; Li et al., 2020). This cluster also showed enrichment for genes related to cell wall modification, consistent with the prominent changes in cell wall composition as the fruit ripens and softens. Cluster 10 showed a nearly opposite pattern to cluster 4, with low expression in later fruit development, and high to moderate expression at Stages 1-3 (**Figure 1D**). These earlier stages of fruit development include bursts of cell division and DNA replication and this cluster contained significant hits for DNA replication, nucleotide biosynthesis and several cell wall biosynthetic terms (**Figure 1E**).

### Wild and cultivated tomato show subtle differences in expression patterns

One of the most notable effects of artificial selection between cultivated and wild tomato is fruit size. As the pericarp makes up a substantial portion of the fruit, we wanted to know the extent to which pericarp gene expression patterns differ between the two species. We therefore called differentially expressed genes with a model that included the species as a covariate and used a likelihood ratio test to determine which genes showed a statistically significant difference in gene expression pattern between the two species. The resulting 1,472 genes that exhibited divergent expression patterns between cultivated and wild tomato showed GO term enrichment for plant-type cell wall organisation and lipid biosynthetic processes, with 11 genes assigned to each term, the maximum number of genes for any GO term in this analysis (**Figure 1F**). This enrichment likely reflects both the different flavour profiles of the two fruits as well as their conspicuous differences in pericarp size. A clustering and GO analysis of these 1,472 genes produced clusters with only very subtle differences in gene expression profiles between species and no apparently informative GO terms (**Supplementary Data Figure S2**). Potentially the differences in fruit phenotype between wild and cultivated tomato involve a small number of genes with slight changes in expression pattern, but we cannot rule out that these differences involve changes in timing or expression domains that were not included in our sampling regime.

### Divergence in expression of ethylene and secondary metabolite synthesis genes following domestication

**Figure 2:**
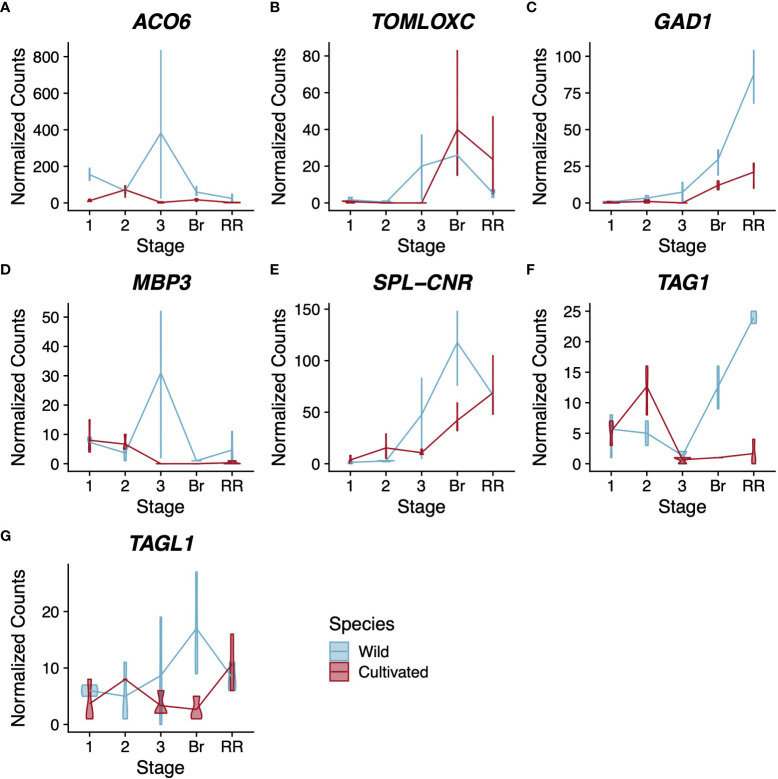
Expression profiles for ethylene-related **(A)**, flavour compound-related **(B–D)**, and regulatory **(D–G)** genes. Normalised counts of gene expression are represented by violin plots. Genes with statistically significant (FDR<0.01) differential expression across stages are shown in bold. Wild tomato is shown in blue and cultivated in red. Stages of fruit development on the X-axis are numbered sequentially followed by “Br” for breaker stage and “RR” for red ripe stage Note that panels have independent Y-axis to maximise readability.

Because cultivated tomato is routinely used as a model to study climacteric fruit ripening, many genes have been identified as playing a role in this process. We asked to what extent the expression patterns of these well-studied ripening genes have changed following domestication. We used our combined wild and cultivated tomato dataset to examine the expression of 21 structural genes involved in ethylene biosynthesis, pigment production, and flavour compound biosynthesis (**Supplementary Data Figure S3**). Among these structural genes, one ethylene-related gene and two flavour compound-related genes have a pattern of expression with statistically significant differences between cultivated and wild tomato (**Figures 2A-C**).

The gene *ACO6* encodes an ethylene biosynthesis enzyme whose role has not been well characterised during fruit development (Houben and Van de Poel, 2019). In our analysis, *ACO6* was the only structural gene related to ethylene synthesis or perception with a statistically significant difference in expression pattern between the two tomato species (**Figure 2A**). The other genes showed either no statistically significant change in expression across pericarp development or no statistically significant difference in pattern between the two species. In contrast, *SpACO6* has higher expression at every stage we sampled in wild tomato compared to *SlACO6* in cultivated tomato. Additionally, *SlACO6* reaches its maximum expression in cultivated tomato at stage 2, which is characterised primarily by cell division, whereas in wild tomato, peak expression of *SpACO6* is reached at stage 3, which is characterised largely by cell expansion (**Table 1**). The peak at stage 3 was not seen for any other *ACO* homologues, suggesting a divergent role for this enzyme during pericarp development (**Supplementary Data Figures S3A-G**).

*TomLoxC* encodes a lipoxygenase and contributes to desirable flavour in tomato fruit (Chen et al., 2004; Shen et al., 2014). In both species, expression was not detected in stages 1-2 of pericarp development (**Figure 2B**). In wild tomato, *SpTomLoxC* transcripts accumulated to moderate levels at stage 3 and breaker stage pericarps, but dropped to much lower levels in red ripe fruits. In cultivated tomato, however, we did not detect any *SlTomLoxC* transcripts until the breaker stage, where we observed maximum expression. The level dropped slightly at the red ripe stage, but still remained higher than the peak expression seen in wild tomato. Polymorphism in *TomLoxC* expression was recently observed in a large study of wild and cultivated tomato accessions, and found to correlate with a large deletion in the promoter of *TomLoxC* that was selected against during domestication (Gao et al., 2019).

Finally, *GAD1* encodes one of three known tomato glutamate decarboxylases, which are responsible for the production of γ-aminobutyric acid (GABA) (Akihiro et al., 2008). In our analysis both tomato species displayed a similar trend for *GAD1* expression during pericarp development, which was consistent with previous studies (Akihiro et al., 2008) (**Figure 2C**). However the two species showed a statistically significant difference in the magnitude of expression, with wild tomato showing approximately 3x higher peak expression of *SpGAD1* at the red ripe stage. GABA can accumulate to very high levels in tomato fruit and is thought to be involved with stress responses and defence (Bouché et al., 2003; MacGregor et al., 2003). Given that wild tomato is a widely recognized resource for introgression of stress tolerance, this difference in a key GABA biosynthesis enzyme represents a potential future avenue for plant breeders (Razali et al., 2018).

### Fruit size-, firmness-, and lignification-related transcription factors differ in expression between wild and cultivated tomato

Because changes in the expression of transcription factors can influence the expression of many target genes simultaneously, we wanted to know the extent to which such regulatory genes differed in expression pattern between these two species. We selected 18 transcription factors with prominent roles in fruit and flower development and used our combined wild and cultivated tomato data set to ask if any of these genes showed statistically significant differences in expression between the two species (**Supplementary Data Figure S4**).

Although many of the selected genes showed statistically significant differential expression across pericarp development with a pattern common to both species, only four had statistically significant support for a difference in expression between the two species. This included three type-II MADS-box genes *MBP3*, *TAG1*, and *TAGL1*, along with the *SQUAMOSA* promoter-binding protein-like transcription factor, *SPL-CNR* (**Figures 2D-G**).

*MBP3* and *AGL11* are orthologous to the Arabidopsis gene *SEEDSTICK*, which helps specify ovule identity (Pinyopich et al., 2003; Ocarez and Mejía, 2016). *AGL11* does not show statistically significant differential expression between tomato species; however its paralog, *MBP3*, does (**Supplementary Data Figures S4A**, **S2D**). Our dataset shows that in cultivated tomato, *SlMBP3* expression is low in stages 1 and 2 before becoming nearly undetectable for the rest of fruit development. In contrast, wild tomato *SpMBP3* is similar to cultivated tomato in expression at stages 1 and 2 but peaks at stage 3 with a roughly 3-fold increase compared to stage 1. Several functional characterizations suggest that *AGL11* helps specify ovule identity in tomato, but we could find no functional characterizations of *MBP3* (Ocarez and Mejía, 2016; Huang et al., 2017).

The tomato genes *TAG1* and *TAGL1* are orthologs of the Arabidopsis genes *AGAMOUS* and *SHATTERPROOF1/2*, respectively (Pnueli et al., 1994; Pan et al., 2010). Both tomato genes have been shown to control several aspects of fruit development and to help specify the identity of stamens and carpels (Pan et al., 2010; Gimenez et al., 2016). Comparing wild and cultivated tomato, *TAG1* shows a more extreme difference in expression than *TAGL1*, though both are statistically significant (p<<0.01, **Figures 2F, G**). In wild tomato, *SpTAG1* expression increases linearly nearly 25-fold between stage 3 and the red ripe stage; however, in cultivated tomato the increase in *SlTAG1* transcripts is barely detectable. For *TAGL1* the departure in expression is more subtle but most obvious at the breaker stage where wild tomato *SpTAGL1* expression peaks and cultivated tomato *SlTAGL1* expression is at its lowest levels. Previous silencing experiments in cultivated tomato suggest that both genes contribute positively to pericarp thickness (Gimenez et al., 2016). Our result is therefore counterintuitive as cultivated tomato generally has a thicker pericarp than wild tomato, but wild tomato showed consistently higher expression of both genes in the pericarp.

The *SQUAMOSA* promoter-binding protein-like transcription factor *SPL-CNR* is thought to be the causative gene for the *Cnr* mutation that affects ripe tomato fruit colour and firmness (Thompson et al., 1999; Eriksson et al., 2004; Manning et al., 2006; Lai et al., 2020). In our analysis, *SPL-CNR* showed a statistically significant difference in expression between the two tomato species (*p*=3.2x10^-4^) with wild tomato showing higher expression in both stage 3 and breaker stage pericarps (**Figure 2E**). Recently *SPL-CNR* expression has been shown to negatively affect cell-to-cell adhesion and to promote cell death (Lai et al., 2020), consistent with a model whereby low expression of *SPL-CNR* in the *Cnr* mutant could lead to a non-softening fruit due to increased cell adhesion or lower levels of cell death. The decreased firmness in mature wild tomato fruits coupled with their higher expression of *SlSPL-CNR* and the increased desirability of firmer cultivated tomato fruits suggests that the expression changes at the *SlSPL-CNR* locus could have been the result of domestication (Tanksley et al., 1996; Doganlar et al., 2002).

### Desert tobacco pericarp transcriptome is enriched for secondary metabolite synthesis and shows fewer differentially expressed genes than tomato

In contrast to tomato, desert tobacco (*Nicotiana obtusifolia*) produces a dry capsular fruit. We extracted RNA from pericarps at stages 1-3 as well as a “transition” stage as the fruit is maturing, analogous to breaker stage in tomato (**Table 1**). Physiologically mature desert tobacco fruits are dry and highly lignified, and we were unable to extract RNA from this final stage.

**Figure 3:**
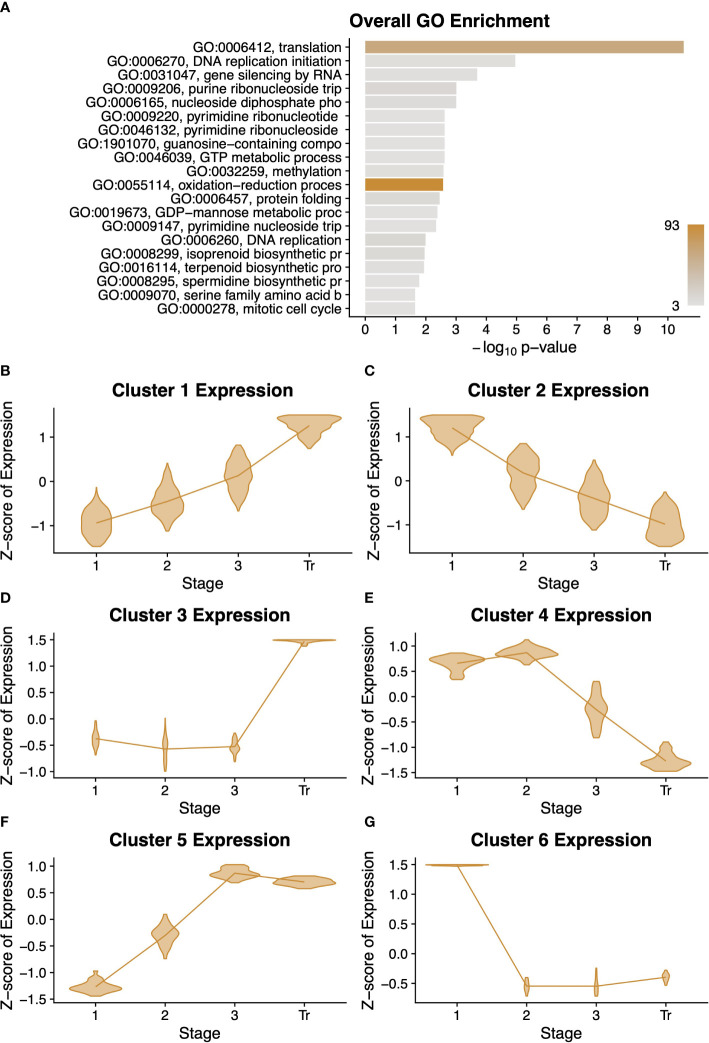
Summary of desert tobacco differentially expressed genes. A gene ontology (GO) term enrichment analysis **(A)** performed on all 1351 differentially expressed genes. All clusters of differentially expressed genes are described with violin plots of normalised expression at each stage of development **(B–G)** comprising. Stages of fruit development in the axis of **(B–G)** are numbered sequentially followed by “Tr” for transition to mature stage.

Because fruit development in desert tobacco has not been molecularly characterised, we examined gene expression dynamics in desert tobacco pericarp development. We applied a similar model that required the expression of a gene be statistically significantly different between at least two stages in order to be considered differentially expressed. We uncovered 1,392 desert tobacco genes with differential expression across the four stages, much fewer than the 6,165 differentially expressed genes among the tomato stages. We performed a GO analysis on this cohort of genes and found that they largely relate to either DNA replication and synthesis or to the synthesis of secondary metabolites such as spermidine or terpenoids (**Figure 3A**). Interestingly, the set of genes with conserved expression among the two tomato species also showed an enrichment for secondary metabolites including the polyamine spermidine (**Figure 1**).

We performed an analysis to sort the differentially expressed genes into clusters with similar expression profiles over time. This unsupervised method produced six profiles, and for each profile we performed a GO analysis (**Figures 3B-G** and **Supplementary Data Figure S5**). Interestingly, clusters 1, 3, and 5 have roughly complementary patterns to clusters 2, 6, and 4, respectively. Clusters 1 and 3 both contain several terms related to protein modification or degradation, while cluster 5 is primarily enriched for lipid and fatty acid biosynthesis. Clusters 2, 4, and 6 generally have a pattern of decreasing expression over time, and these clusters are all enriched for very basic metabolic functions such as DNA replication, translation, and biological processes. This decrease in expression could reflect the beginning of senescence and a general cessation of active metabolic processes.

### Solanaceae expression patterns align with prominent developmental processes

The tomato species differ in fruit type from desert tobacco, and we wanted to know the extent to which expression patterns are conserved (or not) among the fruit of these phenotypically diverse, but relatively closely related taxa. To answer this we used OrthoFinder2 to find single-copy orthologous genes from dry-fruited desert tobacco and both fleshy-fruited tomato species together (Emms and Kelly, 2019). Because we were unable to extract RNA from mature desert tobacco capsules, these datasets are sampled at four comparable developmental stages (**Table 1**). We then applied two nested statistical models to test for differential expression over time that was conserved among all species or divergent between fruit types.

**Figure 4:**
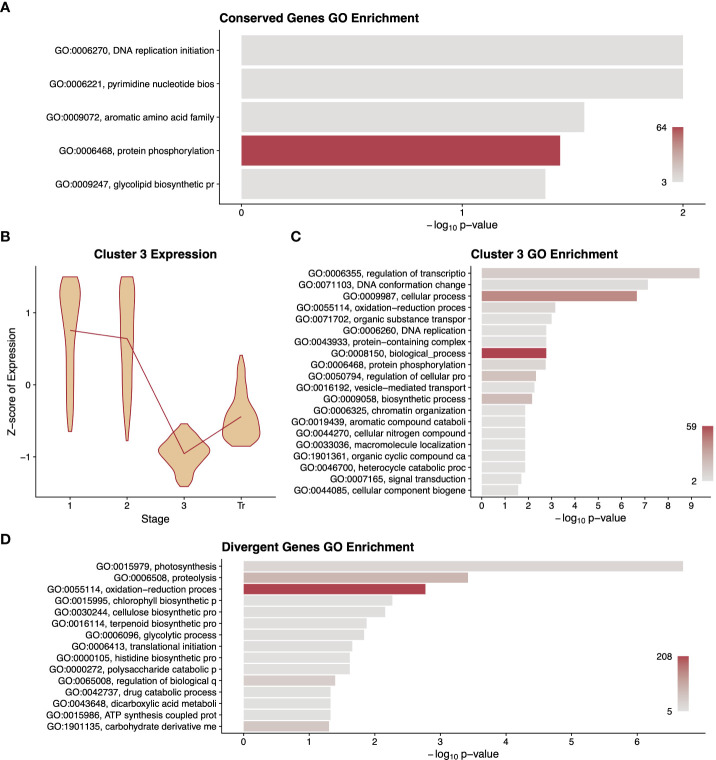
Summary of differentially expressed orthologous genes. A gene ontology (GO) term enrichment analysis **(A)** performed on differentially expressed genes that had conserved patterns among the three species. A representative cluster of 796 differentially expressed genes conserved among species is described with violin plots of normalised expression at each stage of development **(B)** along with a GO enrichment analysis **(C)** of the genes in that cluster. A gene ontology (GO) term enrichment analysis **(D)** performed on differentially expressed genes that had different patterns between fruit types. GO term descriptions to the left of the enrichment graphs are truncated for space and sorted by p-value. The bars are colored by the number of genes assigned to each GO term with legends in the lower right of the graph. Stages of fruit development in the axis of B-GD are numbered sequentially followed by “Tr” for transition to mature stage.

Only 1,235 single-copy orthologs showed a statistically significant conservation of expression pattern across all three species. As a cohort, this comparatively small number of genes was enriched for five GO terms, including DNA replication and protein phosphorylation (**Figure 4A**). To examine finer scale patterns among these genes, we performed unsupervised clustering followed by a GO analysis of the genes in each cluster. This revealed seven profiles of gene expression patterns over time (**Supplementary Data Figure S6**). The expression patterns and GO term enrichments for the clusters largely agree with prominent developmental processes at various stages. For instance, cluster 3 has highest expression at stages 1 and 2 and is enriched for several terms related to DNA replication, which is known to occur early in fruit development (**Figures 4B, C**) (Gillaspy et al., 1993; Tanksley, 2004; Pabón-Mora and Litt, 2011).

Our search for single-copy orthologs that have statistically significant differences in expression pattern between fruit types yielded 4,647 genes. A GO term analysis of this set of genes revealed terms underlying known phenotypic differences between these two fruit types including terpenoid biosynthetic processes, which are likely related to flavour compound production, as well as polysaccharide catabolism, cellulose biosynthesis, glycolytic processes, and carbohydrate derivative metabolism, which could relate to the differential accumulation of sugars and/or cell wall composition between these fruit types (**Figure 4D**). Unsupervised clustering and GO analyses were also carried out on this dataset; however, this did not yield readily informative patterns or terms (**Supplementary Data Figure S7**).

### Solanaceae orthologs of ripening-related genes show fruit type-specific expression patterns

Given the interesting differences between wild and cultivated tomato in expression of the ripening related structural and regulatory genes, we asked to what extent the expression pattern of these genes has diverged between the fleshy-fruited tomato species and the dry-fruited desert tobacco. We restricted our analysis to genes that had a single unambiguous ortholog in all three species and found orthologs for four of 12 ethylene-related structural genes and five of 18 transcription factors (**Table 2**). We then pooled replicates from both tomato species as a single representative fleshy-fruited taxon and contrasted their expression values with those from desert tobacco. This effectively averages differences in expression that may have been apparent between wild and cultivated tomato but allows us to search for genes with strong signal of fruit-type specific expression over time. Using a likelihood ratio test, we were able to discern if the expression patterns show conservation between fruit types, within fruit types, or are divergent between fruit types.

**Table 2:** Table showing the relationships between orthologous genes identified in this study.

Orthology and Abbreviations			
Gene Name	*Solanum* Gene ID	*Nicotiana* Ortholog ID*^1^*	*Nicotiana* Abbreviation
*ACO1*	*Solyc07g049530.3.1*	*—*	*—*
*ACO2*	*Solyc12g005940.2.1*	*—*	*—*
*ACO3*	*Solyc07g049550.3.1*	*—*	*—*
*ACO4*	*Solyc02g081190.4.1*	*NIOBTv3_g13660.t1*	*NoACO4*
*ACO5*	*Solyc07g026650.3.1*	*NIOBTv3_g38689.t1*	*NoACO5*
*ACO6*	*Solyc02g036350.3.1*	*NIOBTv3_g02352.t1*	*NoACO6*
*ACO7*	*Solyc06g060070.3.1*	*—*	*—*
*ACS2*	*Solyc01g095080.3.1*	*—*	*—*
*ACS4*	*Solyc05g050010.3.1*	*—*	*—*
*AGL11*	*Solyc11g028020.3.1*	*NIOBTv3_g14436.t1*	*NoAGL11*
*Cel2*	*Solyc09g010210.3.1*	*NIOBTv3_g19880.t1*	*NoCel2*
*Cel3*	*Solyc07g005840.2.1*	*NIOBTv3_g12440.t1*	*NoCel3*
*CHS-1*	*Solyc09g091510.3.1*	*—*	*—*
*CHS-2*	*Solyc05g053550.3.1*	*—*	*—*
*CTOMT1*	*Solyc10g005060.4.1*	*—*	*—*
*EIL1*	*Solyc06g073720.3.1*	*—*	*—*
*EIL2*	*Solyc01g009170.4.1*	*—*	*—*
*EIL4*	*Solyc06g073730.2.1*	*—*	*—*
*EJ2/MADS1*	*Solyc03g114840.3.1*	*—*	*—*
*EXP1*	*Solyc06g051800.3.1*	*NIOBTv3_g17210.t1*	*NoEXP*
*FUL1*	*Solyc06g069430.3.1*	*NIOBTv3_g28929-D2.t1*	*NoFUL1*
*FUL2*	*Solyc03g114830.3.1*	*NIOBTv3_g39464.t1*	*NoFUL2*
*FYFL*	*Solyc03g006830.3.1*	*NIOBTv3_g10096.t1*	*NoFYFL*
*GAD1*	*Solyc03g098240.3.1*	*NIOBTv3_g11084.t1*	*NoGAD1*
*GAD2*	*Solyc11g011920.2.1*	*—*	*—*
*GAD3*	*Solyc01g005000.3.1*	*—*	*—*
*J*	*Solyc11g010570.2.1*	*—*	*—*
*J2*	*Solyc12g038510.2.1*	*NIOBTv3_g15806.t1*	*NoJ2*
*MADS-RIN*	*Solyc05g012020.4.1*	*—*	*—*
*MBP10*	*Solyc02g065730.2.1*	*NIOBTv3_g07845.t1*	*NoMBP10*
*MBP20*	*Solyc02g089210.4.1*	*NIOBT_gMBP20.t1*	*NoMBP20*
*MBP3*	*Solyc06g064840.4.1*	*—*	*—*
*MC*	*Solyc05g056620.2.1*	*NIOBTv3_g18077.t1*	*NoMC*
*NAC-NOR*	*Solyc10g006880.3.1*	*NIOBTv3_g08302.t1*	*NoNOR*
*NR/ETR3*	*Solyc09g075440.4.1*	*NIOBTv3_g10291.t1*	*NoETR3*
*PGA2A*	*Solyc10g080210.2.1*	*—*	*—*
*PL1*	*Solyc03g111690.4.1*	*—*	*—*
*PSY1*	*Solyc03g031860.3.1*	*NIOBTv3_g17569.t1*	*NoPSY1*
*Solyc03g117740.3.1*	*Solyc03g117740.3.1*	*NIOBTv3_g22270.t1*	*NIOBTv3_g22270.t1*
*Solyc04g072038.1.1*	*Solyc04g072038.1.1*	*NIOBTv3_g10008.t1*	*NIOBTv3_g10008.t1*
*Solyc06g065310.3.1*	*Solyc06g065310.3.1*	*NIOBTv3_g12238.t1*	*NIOBTv3_g12238.t1*
*Solyc07g064300.3.1*	*Solyc07g064300.3.1*	*NIOBTv3_g11662.t1*	*NIOBTv3_g11662.t1*
*SPL-CNR*	*Solyc02g077920.4.1*	*NIOBTv3_g27953.t1*	*NoSPL-CNR*
*STM3*	*Solyc01g092950.3.1*	*—*	*—*
*TAG1*	*Solyc02g071730.4.1*	*NIOBTv3_g22632-D2.t1*	*NoAG*
*TAGL1*	*Solyc07g055920.4.1*	*NIOBTv3_g13969.t1*	*NoSHP*
*TM29*	*Solyc02g089200.4.1*	*NIOBTv3_g14235.t1*	*NoSEP1*
*TM3*	*Solyc01g093965.2.1*	*—*	*—*
*TM5*	*Solyc05g015750.3.1*	*—*	*—*
*TOMLOXC*	*Solyc01g006540.4.1*	*—*	*—*

^1^Only one-to-one and many-to-one orthologs.

Each row represents a single gene of interest with its abbreviated gene name in the first column, tomato gene identifier in the second column, desert tobacco gene identifier (if known) in the third column, and the desert tobacco abbreviated gene name (if known) in the fourth column.

**Figure 5:**
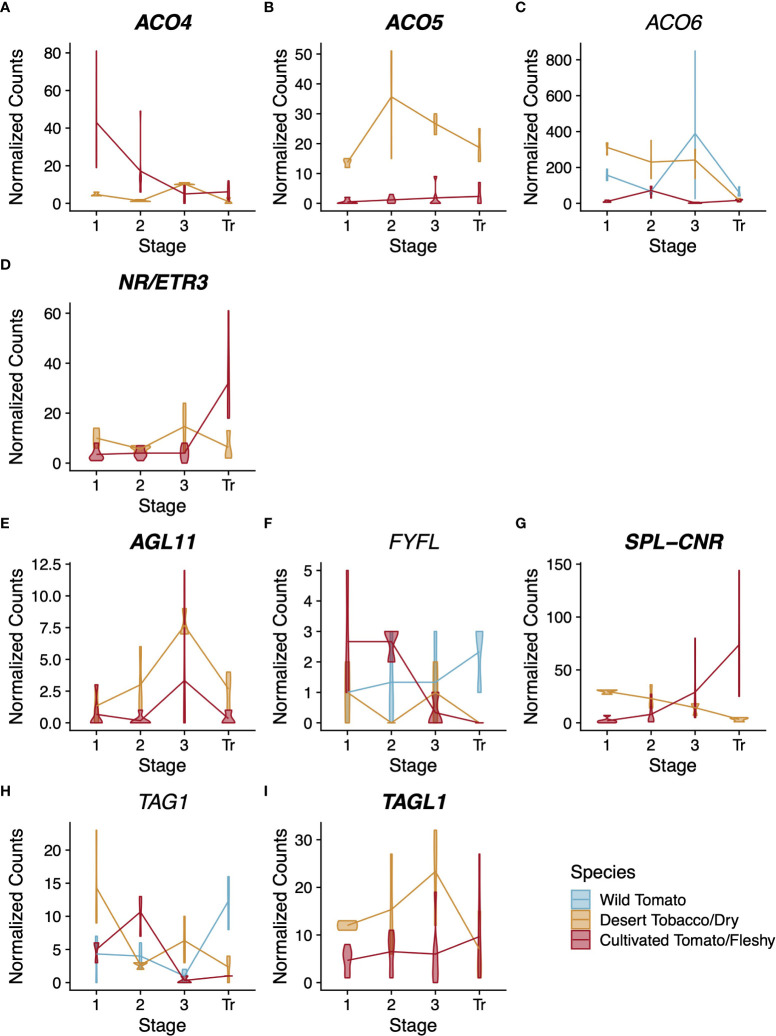
Expression profiles for ethylene-related **(A–D)** and regulatory **(E–I)** genes across the three solanaceous species. Normalised counts of gene expression are represented by violin plots. Genes with statistically significant (FDR<0.01) differential expression across stages are shown in bold. Dry-fruited desert tobacco values are shown in yellow. When the expression pattern is better described by individual species trends (based on a likelihood ratio test), wild tomato violin plots are shown in blue and cultivated tomato plots in red, otherwise both tomato species are shown together in red. Stages of fruit development on the X-axis are numbered sequentially followed by “Tr” for transition to maturity stage. Note that panels have independent Y-axis to maximise readability.

Interestingly, all nine of the genes for which we determined orthology show a decrease in expression between stage 3 and the transition stage of the desert tobacco capsule (**Figure 5**). This result echoes that seen in desert tobacco clusters 2, 4, and 6 from the entire cohort of 1,392 differentially expressed genes, suggesting again that there may be a trend toward gradual ramping down of metabolic processes as the fruit begins to senesce.

Among the ethylene-related structural genes, we found orthologs for *ACO4, ACO5*, *ACO6*, and *NR/ETR3* (**Figures 5A-D**). *ACO4*, *ACO5*, and *NR/ETR3* each have statistically significant differences in their expression patterns between the fruit types (*p*=1.01x10^-9^, 8,9x10^-20^, and 1.8x10^-5^, respectively). *ACO6* is differentially expressed over developmental time but this pattern is different in each of the three species. The lack of conservation for the *ACO6* expression pattern is likely due to the differences in expression among the two tomato species, which have nearly opposite patterns of expression over time. Interestingly, for *ACO5*, all desert tobacco timepoints show higher expression magnitudes than in either tomato species, and for *ACO6* desert tobacco shows higher expression than cultivated tomato. However desert tobacco capsules are non-climacteric fruits, and the high expression of these ethylene biosynthetic genes suggests that the involvement of ethylene in maturity of desert tobacco and other dry fruits deserves further study.

Among the transcription factors, we resolved unambiguous, single-copy orthologs across the three species for *AGL11, FYFL*, *SPL-CNR*, *TAG1*, and *TAGL1* (**Figures 5E-I**). Only *FYFL* and *TAG1* lacked statistically significant conservation of expression pattern among the three species (**Figures 5F, H**). In contrast to our tomato comparisons, *AGL11*, which did not show statistically significant differences between tomato species, does show statistically significant differences between fruit types (**Figure 5E**, *p*=6.5x10^-3^, 5x10^-5^, and 1.7x10^-5^). As mentioned previously, the role of *AGL11* and its paralog *MBP3* in the pericarp is unclear at present, but the statistically significant divergence in expression pattern of *AGL11* between fruit types and of *MBP3* among tomato species highlights the need for further study of these gene functions following their duplication.

Orthologs of *SPL-CNR* and *TAGL1* both showed statistically significant conservation in their expression patterns by fruit types (**Figures 5G, H**, *p*=5.4x10^-17^ and 5.6x10^-3^). The Arabidopsis ortholog of *TAGL1* promotes the formation of the dehiscence zone in the pericarp of that dry fruit (Ferrándiz et al., 2000). In our analysis, the pattern of expression for *TAGL1* is higher overall in dry fruited species and peaks at stage 3 as the dehiscence zone is forming. This provides some evidence for the functional conservation of this gene’s role in dry fruit dehiscence. For *SPL-CNR*, we observe roughly opposing patterns of expression between dry and fleshy fruits. *SPL-CNR* increases in expression as fleshy fruits enter the breaker stage, before they have begun to soften. In contrast, we see a decrease in *SPL-CNR* expression as dry fruits approach dehiscence. Additional functional studies of this gene’s role across dry-fruited species could help extend its established role in cell-cell adhesion and clarify its potential role in dry fruit maturity.

### A small set of genes show conservation of expression pattern between dry and fleshy fruit

Our analysis of the tomato species and desert tobacco revealed a number of informative patterns, but all three species belong to the same family. As a result, we cannot tell if common patterns of gene expression are due to shared phylogenetic history or represent trends across angiosperm fruit development. We wanted to find generalizable trends in gene expression that might underlie the divergence between dry and fleshy fruit development or support conservation of certain gene expression patterns between these two phenotypically diverse fruits. We therefore chose to add *Arabidopsis thaliana*, which produces a dry silique and melon (*Cucumis melo*), which produces a type of berry with a leathery rind known as a pepo.

**Figure 6:**
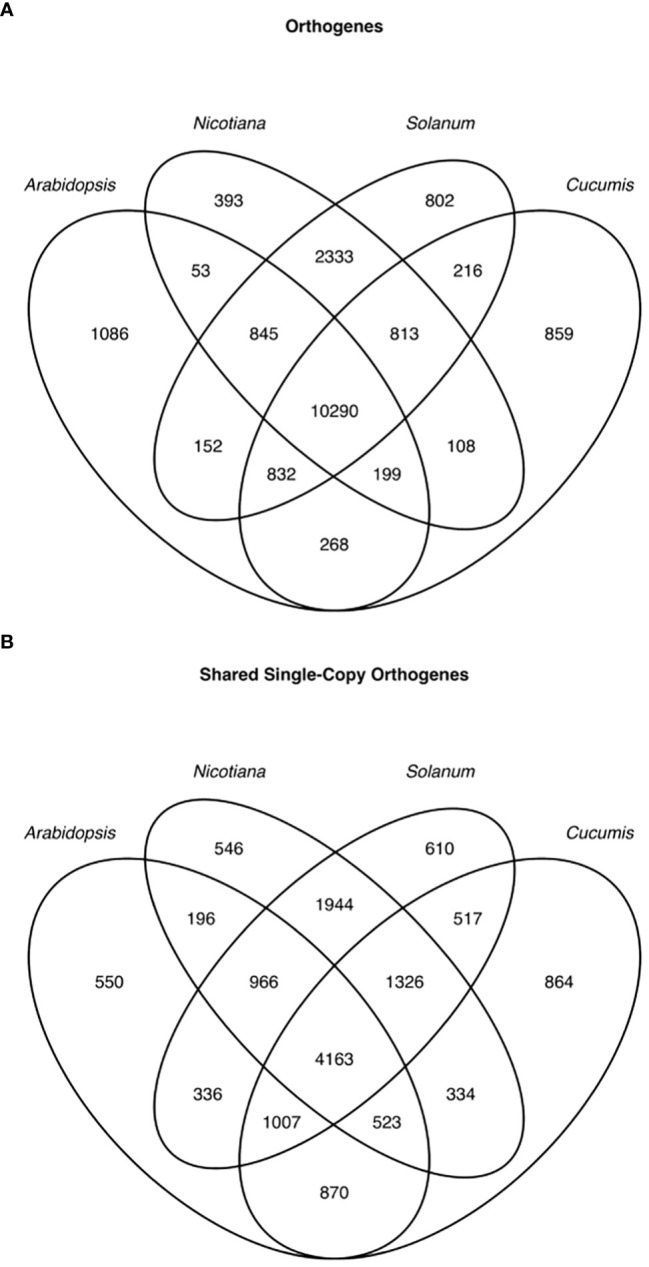
Venn diagram of orthologous genes (orthogenes) among the 4 genera used in this study. All genes across the 4 genera **(A)** and only single-copy genes **(B)**.

In order to enable expression comparisons between and among species, we used Orthofinder2 to group genes from these species into orthologous groups based on protein sequence similarity and phylogenetic relationships (Emms and Kelly, 2019). Due to their high degree of similarity, and because we had mapped wild tomato RNAseq using the cultivated tomato genome, we used cultivated tomato protein sequences in the orthology search to represent both cultivated and wild tomato. For subsequent gene expression analyses, however, the two tomato species were not combined. We were able to group the genes from these species into 19,249 orthogroups (**Figure 6A**); however, many orthogroups were not shared among all species, and even among universally shared orthogroups, there were many cases of gene family expansion or loss within a single species. Because comparing transcript levels among unequal numbers of genes across species is not meaningful, we limited our interspecific expression analysis to only single-copy genes falling into universally present orthogroups. This filtering left 4,163 orthogenes for comparisons among both tomato species, desert tobacco, Arabidopsis, and melon (**Figure 6B**).

For these five species, we wanted to use comparable developmental stages to see if any orthologous genes shared similar expression dynamics over time among all species or among species with similar fruit types. After integrating the publically available Arabidopsis and melon pericarp RNAseq data with our own tomato and desert tobacco datasets, we had comparable data for stage 2, stage 3, and transition stage in all species (**Table 1**).

**Figure 7:**
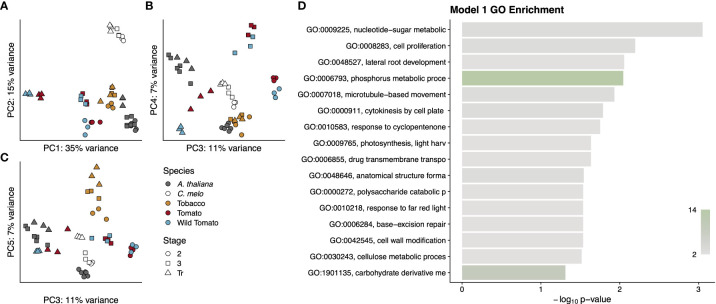
Summary of genes from Model 1. Principal components analysis **(A–C)** of gene expression values for each RNA-seq library. Points are colored by species and shaped by developmental stage as indicated in the legend. Principal components used for each graph are indicated on the axis along with the proportion of variance explained. A GO analysis **(D)** for the entire cohort of genes. GO term names to the left of the graph are truncated to available space. Terms are sorted by p-value, which is indicated by the bar height. Bars are colored by the number of genes annotated to that term, as indicated by the colour scale in the lower right.

We first assessed the extent to which any of the 4,163 orthologous genes were differentially expressed over time and shared a conserved pattern across all five species. To call differential expression across the three stages, we used a model (Model 1) that is blind to species but requires a gene to have a statistically significant change in expression between at least two stages in order to be differentially expressed. Surprisingly, this resulted in only 121 orthologous genes with a pattern of differential expression over time that is the same in all 5 species (**Supplementary Data File S1**). To determine if the expression data from these genes showed a detectable signal based on developmental stage, species, or fruit type, we conducted a principal component analysis using their expression values (**Figures 7A-C**). Model 1 did not consider species in calling differentially expressed genes, and in fact the variance explained by the first five principal components (PC) appears not to have strong signal for interspecific differences. The notable exception to this is PC2, which explains 15% of the variance and seems mostly to separate melon from the other four species; however, PC2 also separates stage 2 from later stages in both tomato species as well as stages 2 and 3 from the transition stage in Arabidopsis. PC1 explains 35% of the variance and largely distinguishes the breaker stage tomato samples from all other samples. PC3 serves to differentiate the three developmental stages of tomato from one another and also separates stage 2 samples from later stages in Arabidopsis. The developmental stages of melon are weakly distinguished by PC4 and more prominently by PC5, each of which explain 7% of the variance. PC5 also weakly separates the developmental stages of desert tobacco.

To categorise these 121 genes, we performed a GO term enrichment analysis and found a number of terms relating to prominent processes common across fruit development including cell proliferation, anatomical structure formation, cytokinesis, and cell wall modification (**Figure 7D** and **Supplementary Data Figure S8**). The shared expression patterns among these 121 genes showed only two clusters of expression profiles. Cluster 1 contains genes whose expression increases between stage 3 and the transition stage, while cluster 2 contains genes whose expression is generally decreasing during fruit development. The genes in cluster 1 are predicted to function in nucleotide metabolism, membrane and organelle structure and processes, lipid and carbohydrate metabolism, ion transport, and similar cellular processes that cannot be easily tied to any specific developmental outcomes. Given that these were pericarp transcriptomes, this is to be expected, because the pericarps of fleshy and dry fruit have little in common other than basic cellular processes like cell division and cellular metabolic processes. The features that distinguish dry and fleshy fruit, such as lignification of the former or cell softening of the latter, are likely to involve very different pathways. Cluster 2 genes are enriched for GO terms relating to DNA replication, cell division, and the phragmoplast, which forms late during cytokinesis and patterns the nascent cell wall. Enrichment for these terms is consistent with the observed cell divisions during stages 1 and 2 and the decline of cell division as fruit development proceeds into stages 3 and 4. This cluster also shows enrichment for several terms related to the thylakoid membrane of the chloroplast, which could be related to the developmental transition of fruits from photosynthetic sources to sinks.

The very small number of genes with conserved patterns across all five angiosperm species further suggests that it may be possible to define a core set of pericarp development-related genes that have a conserved function despite large divergences in both evolutionary time and in phenotype.

### Divergence in expression of genes related to cell division, plastid localization, and secondary cell wall composition between dry and fleshy fruits

Having established that few orthologous genes have conserved expression patterns across all five species, we next asked if and to what extent genes might show conservation of expression patterns within, but not between, fruit types. We reasoned that these fruit-type specific patterns could shed light on developmental processes shared by evolutionarily distant species with a common phenotype, dry or fleshy fruits. To answer this question, we created a model to call differentially expressed orthologous genes (Model 2) that is aware of fruit type for each of the five species but is blind to the species themselves. Like Model 1, which we used to find conserved patterns across all species, Model 2 also requires that a gene have a statistically significant change in expression between at least two of the three developmental stages. Because Models 1 and 2 are nested, genes are only differentially expressed by Model 2 if their expression pattern is better explained by Model 2 than by Model 1, as determined by a likelihood ratio test. This ensures that the difference in fruit type is driving the determination of differential expression.

**Figure 8:**
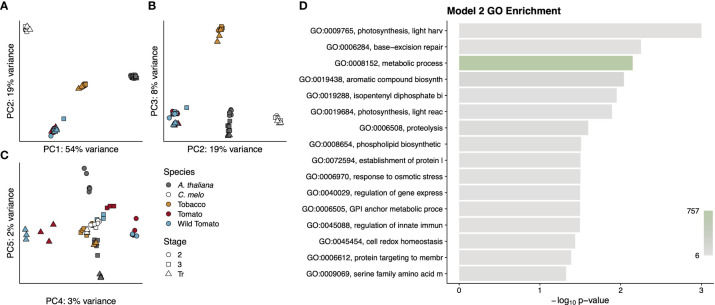
Summary of genes from Model 2. Principal components analysis **(A-C)** of gene expression values for each RNA-seq library. Points are colored by species and shaped by developmental stage as indicated in the legend. Principal components used for each graph are indicated on the axis along with the proportion of variance explained. A GO analysis **(D)** for the entire cohort of genes. GO term names to the left of the graph are truncated to available space. Terms are sorted by p-value, which is indicated by the bar height. Bars are colored by the number of genes annotated to that term, as indicated by the colour scale in the lower right.

Interestingly, Model 2 determined that nearly half of the 4,163 single-copy orthologous genes had divergent patterns of expression between dry and fleshy fruited species (**Supplementary Data File S2**). In contrast, only 202 (<5%) of these single-copy orthologous genes were differentially expressed when comparing between the wild and cultivated tomato species. We performed a principal component analysis to see if any grouping by species, developmental stage, fruit type, or evolutionary distance might be driving this large number of differentially expressed genes (**Figures 8A-C**). In this analysis, the first three principal components, which collectively explained 81% of the variance, served primarily to distinguish among the species. PC1 accounted for the majority of the variance (54%) and separated the dry and fleshy fruited species. On PC1, desert tobacco was separated from the two tomato species, but not as dramatically as Arabidopsis from melon, suggesting that PC1 might also incorporate some amount of variance due to phylogenetic distance in addition to fruit type. Similarly, PC2, which explained 19% of the variance, did not separate the two dry-fruited species but placed tomato and melon at two extremes. PC2 therefore combined both dry fruits but distinguished between two categories of fleshy fruits. PC3, which accounted for 8% of the variation, only seemed to separate desert tobacco from the other four species. PC4 and PC5 captured 3% and 2% of the variance, respectively, and showed a striking perpendicular separation of developmental stages in tomato and Arabidopsis but placed both melon and desert tobacco at their intersection, roughly overlapping with stage 3 of tomato (**Figure 8C**). Interestingly, in contrast to PC1-3, which primarily separated species, PC4 was the only principal component we examined that was able to separate the two tomato species, and even here the separation was only evident for the breaker stages samples.

To determine what sorts of genes were captured by this model, we performed a GO enrichment on all 1,795 genes (**Figure 8D**). In contrast to the very focused enrichment seen in Model 1, the genes from Model 2 were enriched for more diverse terms. In fact, the enrichment of the very high-level metabolic processes term with 757 associated genes highlights the diversity of functions that separate pericarp development in dry- and fleshy-fruited species. Even lower-level enriched terms fall into very disparate categories such as protein trafficking, secondary metabolite synthesis and regulation of gene expression.

**Figure 9:**
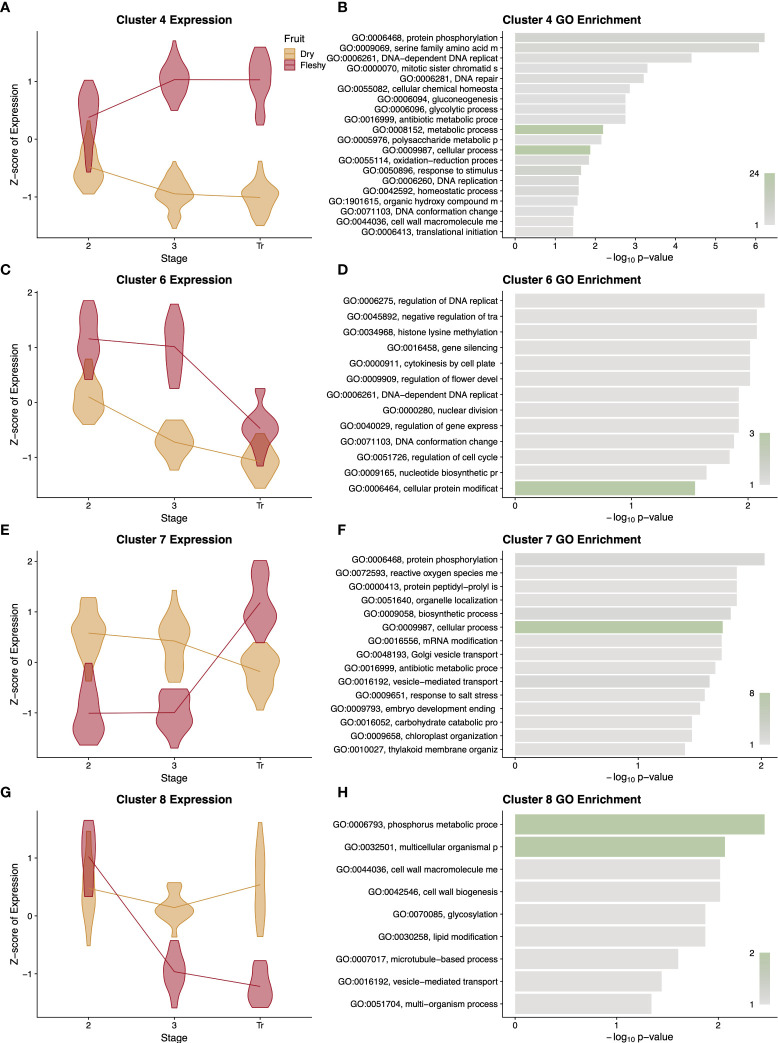
Summary of differentially expressed orthologous genes. Representative clusters of differentially expressed genes with patterns that differ between dry and fleshy fruited taxa are presented with violin plots of normalised expression at each stage of development **(A, C, E, G)** along with a GO enrichment analysis **(B, D, F, H)** of the genes in that cluster. Clusters 4, 6, 7, and 8 comprise 366, 102, 108, and 96 orthologous genes, respectively. GO term descriptions to the left of the enrichment graphs are truncated for space and sorted by p-value. The bars are colored by the number of genes assigned to each GO term with legends in the lower right of the graph. Stages of fruit development in the axis of **(A, C, E, G)** are numbered sequentially followed by “Tr” for transition to mature stage.

Because of the diversity of functional terms in the GO analysis of the entire cohort of genes, we next asked in what ways the patterns of expression diverged between fruit types and what sorts of genes displayed these patterns. Our clustering analysis resulted in eight expression profiles, and we performed a GO analysis on each cluster (**Supplementary Data Figure S9**). Interestingly many, but not all, of these clusters showed distinctive expression profiles with more focused enrichments. In cluster 4 the relative expression diverges over time between dry and fleshy fruits, with fleshy fruits showing higher expression (**Figure 9A**). This cluster was enriched for several terms relating to glucose and polysaccharide synthesis, which could correspond to the accumulation of sugars in fleshy fruits as they begin to ripen (**Figure 9B**). Similarly, in cluster 6, dry fruits show the same pattern as cluster 4, but fleshy fruits show a slight drop in gene expression at stage 3 followed by a larger drop at the transition or breaker stage (**Figure 9C**). This cluster is enriched for terms relating to DNA replication and cytokinesis, likely related to the burst of cell division in stage 2 of fruit development followed by the endoreduplication that occurs in stage 3 of tomato pericarps (**Figure 9D**). At the transition or breaker stage of tomato fruit development, chloroplasts are known to reorganise and convert to chromoplasts, which store the conspicuous red pigments. This process is reflected in cluster 7 where dry fruits slowly drop in expression over time, but fleshy fruits show a jump in expression at the transition stage (**Figure 9E**). This cluster is enriched for a number of terms relating to plastid remodelling and trafficking (**Figure 9F**). Finally, cluster 8 highlights the key feature of dry fruit pericarps, which deposit lignin polymers in their secondary cell walls as they develop. In cluster 8, dry fruit expression remains moderate, while fleshy fruit expression values drop and remain low following stage 2 (**Figure 9G**). GO terms enriched in this cluster include a number of cell wall biogenesis terms (**Figure 9H**). Overall the profiles and enrichments seen in these clusters support a number of hypotheses regarding differential expression developmental processes separating dry and fleshy fruits and provide a basis for more direct studies of function divergence (or conservation) between these diverse fruit types.

## Discussion

Across angiosperm evolution there have been repeated transitions from ancestral dry fruits to derived fleshy fruits, often with dramatic consequences. Although the morphological and developmental basis of these transitions have been well-documented, the underlying molecular and genetic mechanisms that enable or hinder these transitions have received less attention. Here we present evidence for a small set of “core” genes whose patterns of differential expression during pericarp development are conserved across several angiosperm taxa. We also show that a much larger set of “accessory” genes exists with patterns of differential expression during pericarp development that are similar within but different between dry- and fleshy-fruited species. The expression patterns of these core and accessory genes echo a number of phenotypic observations regarding differences in dry and fleshy fruit cell wall composition, cell division, and secondary metabolite production. Interestingly, these expression patterns also raise new questions about the role of ethylene in dry fruit maturity as well as the role of additional transcription factors in dry fruit dehiscence.

At lower taxonomic levels, our data also highlight a number of gene expression differences correlated with the domestication of tomato (*S. lycopersicum*) from its wild ancestor (*S. pimpinellifolium*) and provide further genetic support for previously noted phenotypic differences in fruit size, firmness, and lignification.

We also note that because our conclusions make use of externally generated datasets, and because of the variability in RNA-seq generally, a more thorough expression characterization of the genes highlighted here could be useful to control for variability in growth conditions, sampling times, and other potentially confounding variables.

### Wild and domesticated tomato show differences in the expression of genes regulating domestication-related functions

Although wild and cultivated tomato species share a number of genetic and morphological similarities, cultivated tomato has undergone strong artificial selection (Blanca et al., 2015). The effects of this artificial selection are quite pronounced on the fruits, which are larger, sweeter, and firmer in cultivated than in wild tomato. We detected some potential consequences of this domestication in our pericarp gene expression dataset.

Profiling the expression of 21 ethylene- and flavour compound-related structural genes as well as 18 regulatory genes implicated in fruit ripening, we found a few key differences in expression pattern between wild and cultivated tomato (**Figure 2** and **Supplementary Data Figure S4.3**, and **Supplementary Data Figure S4.4**). The gene *TomLoxC*, which encodes a lipoxygenase, contributes to desirable flavour in tomato fruit and showed different expression patterns between wild and cultivated tomato (Chen et al., 2004; Shen et al., 2014). This locus was previously identified as a target of selection during the domestication of tomato (Gao et al., 2019). The ethylene biosynthesis gene *ACO6* was the only ethylene-related gene in our dataset that showed different patterns of expression between wild and cultivated tomato, with expression of this gene higher at all stages of pericarp development in wild tomato (**Figure 2A**). As we extended our analysis to include the dry-fruited desert tobacco pericarp transcriptome, we also saw comparatively high levels of *NoACO6* expression (**Figure 5C**). In fact, the levels of *NoACO6* expression were higher than in cultivated tomato throughout pericarp development and also higher than wild tomato at Stages 1 and 2, which are characterised by ovary patterning and cell division. We also saw higher expression across pericarp development for another ethylene biosynthetic enzyme *ACO5* in desert tobacco as compared to the two tomato species (**Figure 5B**). Higher expression of ethylene biosynthetic enzymes in this dry fruit is counterintuitive and highlights the need for further study of the roles these specific enzymes, and ethylene more generally, play in the ripening and maturity of dry fruits.

Among the regulatory genes, *MBP3* was expressed at higher levels in wild than cultivated tomato, following the stage of pericarp cell division (**Figure 2D**). The precise role of *MBP3* in tomato is unknown, but its paralog *AGL11* and their mutual ortholog in Arabidopsis both act to specify ovule identity (Pinyopich et al., 2003; Ocarez and Mejía, 2016; Huang et al., 2017). The role of these ovule identity genes in the pericarp is unclear at present, however the grape ortholog of these genes, *VvAGL11*, is adjacent to a QTL that controls both seedlessness and fruit size (Mejía et al., 2011). It could follow then that the differences in *MBP3* expression and in fruit size between wild and cultivated tomato represent possible subfunctionalization following the duplication that produced *AGL11* and *MBP3*.

We also detected species-specific patterns of expression for the transcription factors *TAG1* and *TAGL1* between wild and cultivated tomato (**Figures 2F, G**). Beyond their roles in organ identity, both *TAG1* and *TAGL1* have been shown to contribute positively to pericarp thickness; however, our results show higher expression for these genes in wild tomato, which has a thinner pericarp (Gimenez et al., 2016). Apart from this role in pericarp thickness, numerous orthologs of *TAGL1* are well documented to promote lignification of the pericarp (Ferrándiz et al., 2000; Giménez et al., 2010; Gimenez et al., 2016). We were curious if this difference in *TAGL1* expression between our two tomato species also correlated with changes in expression of structural genes involved in lignin biosynthesis. We queried our results for interspecific expression differences in the first three enzymatic steps of lignin biosynthetic (*SlPAL*: *Solyc09g007920*, *SlC4H*: *Solyc06g150137*, *Sl4CL.1*: *Solyc03g117870*, *Sl4CL.2*: *Solyc06g068650*, and *Sl4CL.3*: *Solyc12g042460*) as well as two enzymes at branch points of the pathway (*SlHCT*: *Solyc03g117600* and *SlF5H*: *Solyc02g084570*). We found that *SlHCT*, the first committed step in the formation of G- and S-type lignin, shows a statistically significant difference in expression pattern between wild and cultivated tomato (p=0.022, likelihood ratio test). This result suggests that, although neither fruit accumulates lignin to substantial levels, there may have been selection against pericarp lignification during tomato domestication. Extending the characterization of *TAGL1* to include desert tobacco, we also saw differences in expression for this gene between fruit types, with higher expression of the desert tobacco *TAGL1* ortholog, *NoSHP* from Stages 1 through 3 of fruit development (**Figure 5I**). This result supports potential conservation of the role *NoSHP* is expected to play in lignin patterning of the dehiscence zones across evolutionarily divergent dry fruits (Ferrándiz et al., 2000).

Finally, we found support for expression differences in *SPL-CNR* between wild and cultivated tomato (**Figure 2E**). Although the pattern of expression for both species shows an upward trend between Stage 2 and Breaker stage, the increase is more dramatic for wild tomato. *SPL-CNR* is believed to be the causative locus underlying the *Colorless non-ripening* (*Cnr*) mutant in tomato (Manning et al., 2006). Disruption of *SPL-CNR* in the *Cnr* mutant results in fruits that fail to soften or undergo colour change at the ripening stage, and this has been related to changes in cell wall composition and cell-cell adhesion (Eriksson et al., 2004; Lai et al., 2020). Although both species of tomato turn red and soften at maturity, that is, neither species displays the extreme *Cnr* phenotype normally, there are quantitative differences in fruit firmness between them. Two large-scale QTL mapping studies of wild and cultivated tomato advanced backcrosses discovered six QTL for fruit firmness, and wild tomato alleles at four of those QTL are shown to decrease fruit firmness (Tanksley et al., 1996; Doganlar et al., 2002). Because soft fruits are more easily damaged during harvest and less desirable to consumers, increasing fruit firmness for cultivated tomato is one target of breeding programs (Barrett et al., 2010). *SPL-CNR* might help increase fruit firmness through its role in cell-cell adhesion, and thus differences in *SPL-CNR* expression between these tomato species could be related to differences in fruit firmness, although many other loci are likely at play. Additionally, the established role of *SPL-CNR* in promoting cell-cell adhesion in tomato has led other authors to speculate that this gene might also play a role in dry fruit dehiscence (Eriksson et al., 2004). If this gene’s function in cell-cell adhesion is conserved among diverse fruit types, then the difference in expression patterns for *SPL-CNR* between fruit types in our analysis is also suggestive of a potential role in dry fruit dehiscence. Including desert tobacco expression data, we observe roughly opposing patterns in *SPL-CNR* expression between dry and fleshy fruits (**Figure 5G**). *SPL-CNR* increases in expression as fleshy fruits enter the breaker stage, before they begin to soften. In contrast, we see a decrease in *SPL-CNR* expression as dry fruits approach dehiscence, where loss of cell adhesion allows the fruit to split open. Additional functional studies of this gene’s role across dry-fruited species could help extend its established role in cell-cell adhesion and clarify confirm its potential role in dry fruit maturity dehiscence and the potential conservation of function across fruits.

In this study, we mapped RNAseq reads from both wild and cultivated tomato to the cultivated tomato reference genome (Hosmani et al., 2019), and in our searches for orthologous genes, we used cultivated tomato sequences as a proxy for both wild and cultivated tomato. This simplified our interspecific comparisons, and mitigated the fact that the genome assemblies of wild tomato are not thoroughly annotated (Razali et al., 2018). Although these decisions enabled better interspecific comparisons, it means we are unable to examine the role of gene duplications and mutations that may have arisen since wild and cultivated tomato split. However, this is unlikely to drastically affect our results since any gene duplications specific to a single species are filtered out of our interspecific comparisons.

### Comparative transcriptome analysis reveals both core conserved fruit development genes, and dry- and fleshy-fruit-specific genes

By examining the expression patterns of single-copy, orthologous genes across wild and cultivated tomato, desert tobacco, Arabidopsis, and melon, we were able to find evidence for two groups of genes in dry and fleshy fruit development, which we have termed the core and accessory genes. The core genes comprise a set of 121 orthologs whose expression patterns in the pericarp are conserved among all five species, while the accessory genome includes 1,795 orthologs whose expression patterns are each similar within fruit types but which show difference between fruit types.

Not all of the 121 core genes have been thoroughly characterised, so at present it is not possible to give a full inventory of functions, but the list suggests common developmental mechanisms that may be necessary for pericarp development. Orthologs for many of these core genes have annotated functions in processes of cell division and cell wall synthesis including the gene *KNOLLE* (*AT1G08560*), which helps pattern the rate and plane of cell divisions (Lukowitz et al., 1996). However other prominent structural genes for cellulose synthase, pectin methylesterase, and pectin lyase, and microtubule organising proteins are also present (*CESA4*, *AT5G44030; PME5*, *AT5G47500; AT5G19730; CORD3, AT4G13370*; *CORD7*, *AT2G31920*; *FUSED*, *AT1G50240*). Other genes in this set have orthologs with annotated function in developmental patterning. For example, the Arabidopsis gene *ARABIDOPSIS CRINKLY4* (*ACR4*, *AT3G59420*) functions in pattern epidermal cells, root asymmetric cell divisions, and cuticle deposition, while *PERIANTHIA* (*AT1G68640*) helps determine floral organ number (Running and Meyerowitz, 1996; Watanabe et al., 2004; De Smet et al., 2008). Beyond the expected cell division and pattern genes we also found several brassinosteroid-related genes as well as *ARGONAUTE7* (AGO7, *AT1G69440*) in this set of core genes. *AGO7* is involved in tasiRNA formation and ultimately helps to regulate development progression from vegetative to reproductive stages as well as leaf morphology in an auxin dependent manner (Adenot et al., 2006; Montgomery et al., 2008). The genes *DWARF4* (*DWF4*, *AT3G50660*) and *TITAN-LIKE* (*TTL, AT4G24900*) are involved in brassinosteroid biosynthesis and growth-responses, respectively (Azpiroz et al., 1998; Lu et al., 2012). The dwarfed phenotype of *dwf4* mutants is related to reduced cell elongation but not cell division, whereas the *ttl* mutant was first characterised based on an endosperm nuclear division defect. The dry and fleshy fruits studied here differ in a number of ways from one another, but overall size, especially in the pericarp tissues we sampled is one very conspicuous difference (Gillaspy et al., 1993). The overall size of a plant organ can be decomposed into the number of cells present and their sizes, so it is interesting that the brassinosteroid related genes in the core set of genes have complementary effects, modulating cell size and nuclear divisions, respectively.

Although our dataset includes eudicot plants from phylogenetically distant families, we believe that the addition of more taxa could help refine this set of core and accessory genes. Because our method is based on patterns among shared, single-copy orthologs however, including additional very distantly related plants or plants with extremely reduced genomes would not be beneficial. We examined patterns of expression for approximately 5,000 orthologs in our five-species comparisons, and this number of orthologs is based not only on the presence of orthologs among all species, but also our ability to confidently identify orthologs. Including more taxa would likely reduce the number of true single-copy orthologs, but because the determination of orthology is based upon finding clusters of proteins with similar sequence and resolving a phylogenetic relationship among them, additional genes could produce more informative gene trees and help increase ortholog numbers.

## Data availability statement

The datasets presented in this study can be found in online repositories. The names of the repository/repositories and accession number(s) can be found below: https://www.ncbi.nlm.nih.gov/, PRJNA646747.

## Author contributions

AR: Conceptualization, Methodology, Software, Formal Analysis, Investigation, Data Curation, Writing, Visualisation. DM: Conceptualization, Methodology. JL: Methodology. AL: Conceptualization, Writing - Review and Editing, Supervision, Project Administration, Funding Acquisition. All authors contributed to the article and approved the submitted version.

## Funding

This work was supported by the National Science Foundation (grant number IOS1456109) and the University of California, Riverside.

## Acknowledgments

We thank the UCR Institute for Integrative Genome Biology (IIGB) Genomics Core for their assistance in various technical aspects of RNAseq library prep and the staff at the UCR High Performance Computing Cluster for their logistical and infrastructural support.

## Conflict of interest

The authors declare that the research was conducted in the absence of any commercial or financial relationships that could be construed as a potential conflict of interest.

## Publisher’s note

All claims expressed in this article are solely those of the authors and do not necessarily represent those of their affiliated organizations, or those of the publisher, the editors and the reviewers. Any product that may be evaluated in this article, or claim that may be made by its manufacturer, is not guaranteed or endorsed by the publisher.

## Supplementary material

Supplementary Figure 1 Summary of clustered gene expression profiles for genes with conserved patterns between wild and cultivated tomato. Violin plots of normalised expression by developmental stage for each cluster are shown on the left and gene ontology (GO) enrichment plots for the genes in the corresponding cluster are shown on the right. GO term descriptions to the left of the enrichment graphs are truncated for space and sorted by p-value. The bars are colored by the number of genes assigned to each GO term with legends in the lower right of each graph. Stages of fruit development in the axis of **(B, D)** are numbered sequentially followed by “Br” for breaker stage and “RR” for red ripe stage.

Supplementary Figure 2 Summary of clustered gene expression profiles for genes with divergent patterns between wild and cultivated tomato. Violin plots of normalised expression by developmental stage for each cluster are shown on the left and gene ontology (GO) enrichment plots for the genes in the corresponding cluster are shown on the right. Profiles for wild tomato are shown in blue, while profiles for cultivated tomato are shown in red. GO term descriptions to the left of the enrichment graphs are truncated for space and sorted by p-value. The bars are colored by the number of genes assigned to each GO term with legends in the lower right of each graph. Stages of fruit development in the axis of **(B, D)** are numbered sequentially followed by “Br” for breaker stage and “RR” for red ripe stage.

Supplementary Figure 3 Summary of clustered gene expression profiles for genes with divergent patterns between wild and cultivated tomato. Violin plots of normalised expression by developmental stage for each cluster are shown on the left and gene ontology (GO) enrichment plots for the genes in the corresponding cluster are shown on the right. Profiles for wild tomato are shown in blue, while profiles for cultivated tomato are shown in red. GO term descriptions to the left of the enrichment graphs are truncated for space and sorted by p-value. The bars are colored by the number of genes assigned to each GO term with legends in the lower right of each graph. Stages of fruit development in the axis of **(B, D)** are numbered sequentially followed by “Br” for breaker stage and “RR” for red ripe stage.

Supplementary Figure 4 Expression profiles for selected regulatory genes. Normalised counts of gene expression are represented by violin plots. Genes with statistically significant (FDR<0.01) differential expression across stages are shown in bold. Where expression pattern is better described by individual species trends (based on a likelihood ratio test), wild tomato violin plots are shown in blue and cultivated tomato plots are shown in red, otherwise the common pattern is shown in red. Stages of fruit development on the X-axis are numbered sequentially followed by “Br” for breaker stage and “RR” for red ripe stage. Note that panels have independent Y-axis to maximise readability.

Supplementary Figure 5 GO Enrichment analysis for desert tobacco gene expression clusters in . GO term descriptions to the left of the enrichment graphs are truncated for space and sorted by p-value. The bars are colored by the number of genes assigned to each GO term with legends in the lower right of each graph. Stages of fruit development in the axis of B-GD are numbered sequentially followed by “Tr” for transition to mature stage.

Supplementary Figure 6 Summary of clustered gene expression profiles for genes with conserved patterns among the three solanaceous species. Violin plots of normalised expression by developmental stage for each cluster are shown on the left and gene ontology (GO) enrichment plots for the genes in the corresponding cluster are shown on the right. GO term descriptions to the left of the enrichment graphs are truncated for space and sorted by p-value. The bars are colored by the number of genes assigned to each GO term with legends in the lower right of each graph. Stages of fruit development in the axis are numbered sequentially followed by “Tr” transition to mature stage.

Supplementary Figure 7 Summary of clustered gene expression profiles for genes with divergent patterns by fruit tpe among the three solanaceous species. Violin plots of normalised expression by developmental stage for each cluster are shown on the left and gene ontology (GO) enrichment plots for the genes in the corresponding cluster are shown on the right. Profiles for dry fruits are shown in yellow, while profiles for both tomato species are shown in red. GO term descriptions to the left of the enrichment graphs are truncated for space and sorted by p-value. The bars are colored by the number of genes assigned to each GO term with legends in the lower right of each graph. Stages of fruit development in the axis are numbered sequentially followed by “Tr” for transition to mature stage.

Supplementary Figure 8 Summary of clustered gene expression profiles for genes with conserved patterns among the five species. Violin plots of normalised expression by developmental stage for each cluster are shown on the left and gene ontology (GO) enrichment plots for the genes in the corresponding cluster are shown on the right. GO term descriptions to the left of the enrichment graphs are truncated for space and sorted by p-value. The bars are colored by the number of genes assigned to each GO term with legends in the lower right of each graph. Stages of fruit development in the axis are numbered sequentially followed by “Tr” transition to mature stage.

Supplementary Figure 9 Summary of differentially expressed orthologous genes. Representative clusters of differentially expressed genes with patterns that differ between dry and fleshy fruited taxa are presented with violin plots of normalised expression at each stage of development along with a GO enrichment analysis of the genes in that cluster. GO term descriptions to the left of the enrichment graphs are truncated for space and sorted by p-value. The bars are colored by the number of genes assigned to each GO term with legends in the lower right of the graph. Stages of fruit development in the axis of **(A, C, E, G)** are numbered sequentially followed by “Tr” for transition to mature stage.

Supplementary File 1 List of gene names for conserved orthologous genes.

Supplementary File 2 List of gene names for divergent orthologous genes.

Supplementary File 3 Mapping statistics for transcriptome data. Columns are the NCBI SRA Accession, species of origin, developmental stage, replicate number, number of mapped reads, percent of reads mapped, mean mapping coverage, percent of mapped reads hitting exons, percent of mapped reads hitting introns, and percent of mapped reads hitting other features.

Supplementary File 4 An Excel file with expression information for the expression profiles plotted in Figures 1, 3, 4, and 9. Data for Figures 1 and 3 show the single gene ID based on the reference annotation, whereas the data for Figure 4 shows both gene IDs in the orthologous pair. In the data for Figure 9 each orthogroup is listed twice, one line for fleshy fruits and another for dry fruits. Only the gene IDs for corresponding fleshy or dry fruited species are shown on a given line, but all genes in the orthogroup were considered orthologous. Expression is shown as a normalized Z-score as determined by DESeq2 and the given model of differential expression testing. Stages listed correspond to the figure labels and cluster identities correspond.
