# Supplementary figures and images for: Multispecies transcriptomes reveal core fruit development genes
Source: Front Plant Sci. 2022 Nov 4;13:954929. doi: 10.3389/fpls.2022.954929 (PMC9673247; doi:10.3389/fpls.2022.954929)

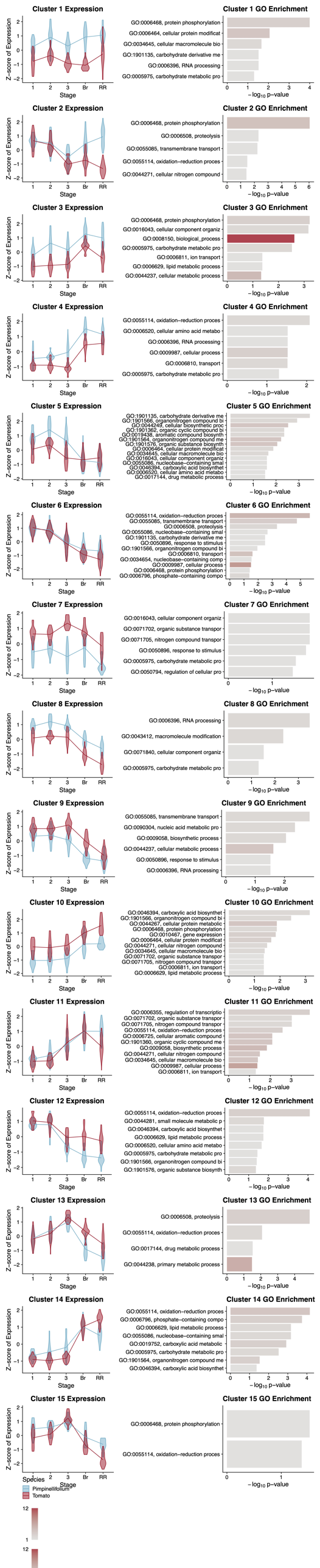

Supplement: Supplementary Figure 2 — Summary of clustered gene expression profiles for genes with divergent patterns between wild and cultivated tomato. Violin plots of normalised expression by developmental stage for each cluster are shown on the left and gene ontology (GO) enrichment plots for the genes in the corresponding cluster are shown on the right. Profiles for wild tomato are shown in blue, while profiles for cultivated tomato are shown in red. GO term descriptions to the left of the enrichment graphs are truncated for space and sorted by p-value. The bars are colored by the number of genes assigned to each GO term with legends in the lower right of each graph. Stages of fruit development in the axis of (B, D) are numbered sequentially followed by “Br” for breaker stage and “RR” for red ripe stage. [file Image_2.jpeg]

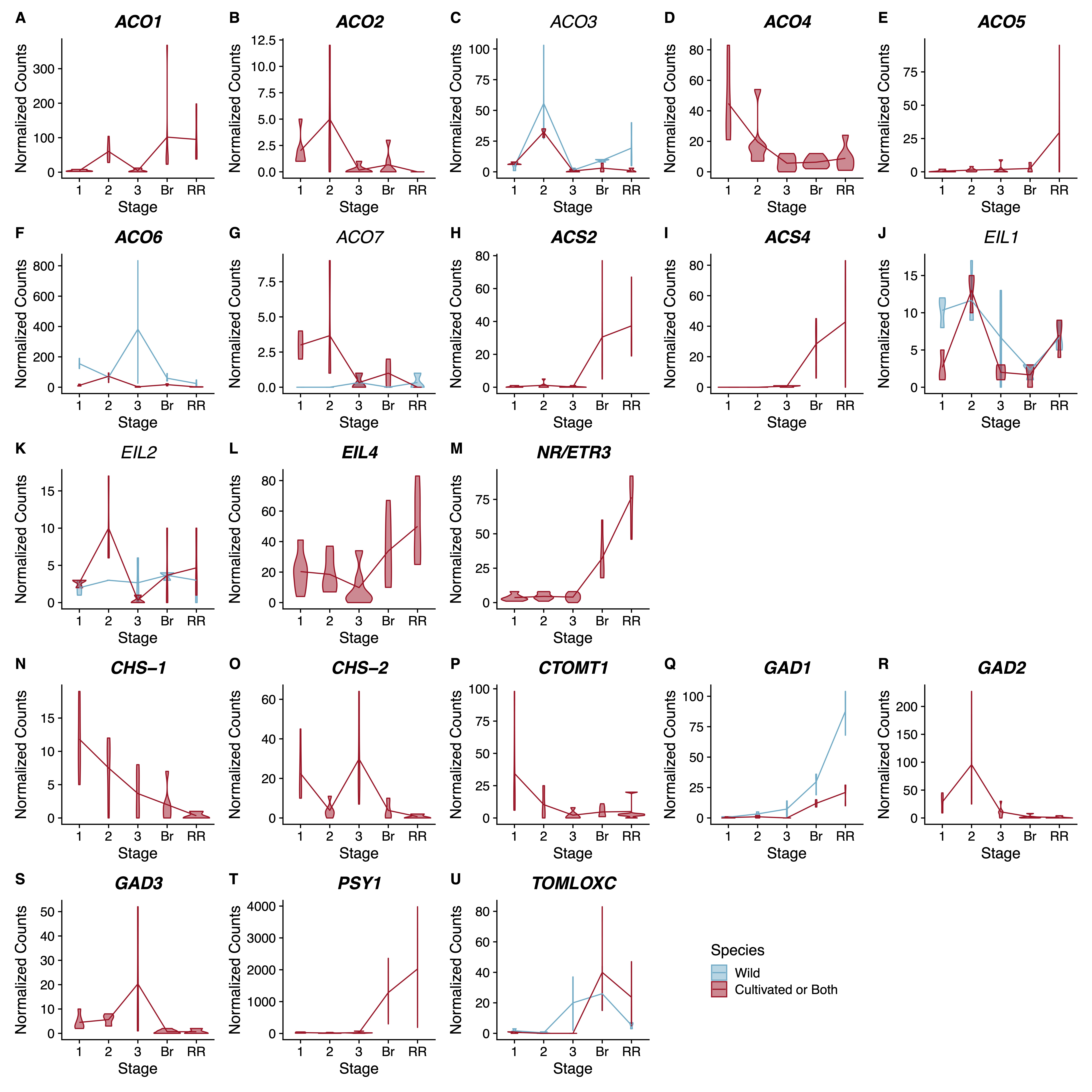

Supplement: Supplementary Figure 3 — Summary of clustered gene expression profiles for genes with divergent patterns between wild and cultivated tomato. Violin plots of normalised expression by developmental stage for each cluster are shown on the left and gene ontology (GO) enrichment plots for the genes in the corresponding cluster are shown on the right. Profiles for wild tomato are shown in blue, while profiles for cultivated tomato are shown in red. GO term descriptions to the left of the enrichment graphs are truncated for space and sorted by p-value. The bars are colored by the number of genes assigned to each GO term with legends in the lower right of each graph. Stages of fruit development in the axis of (B, D) are numbered sequentially followed by “Br” for breaker stage and “RR” for red ripe stage. [file Image_3.jpeg]

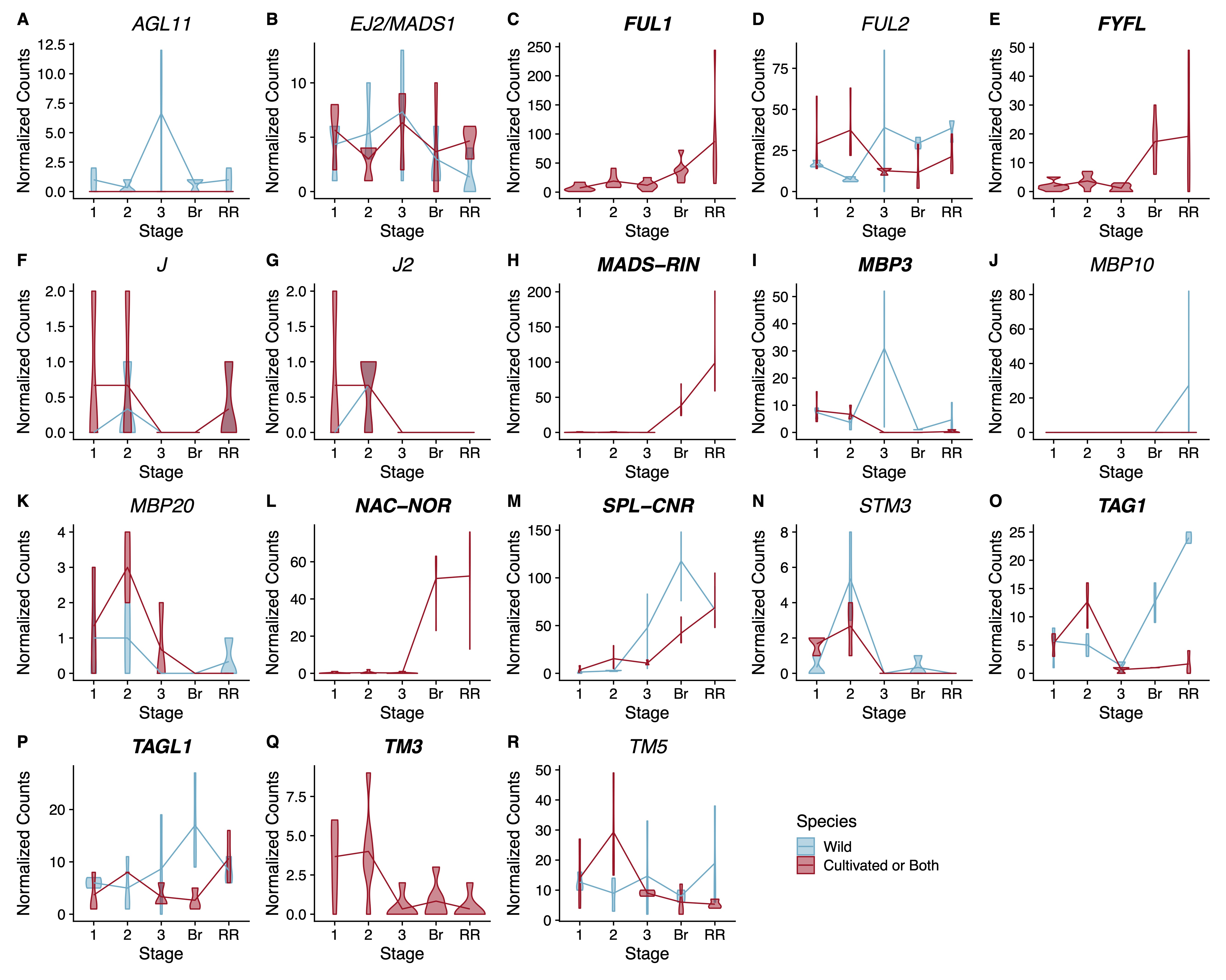

Supplement: Supplementary Figure 4 — Expression profiles for selected regulatory genes. Normalised counts of gene expression are represented by violin plots. Genes with statistically significant (FDR<0.01) differential expression across stages are shown in bold. Where expression pattern is better described by individual species trends (based on a likelihood ratio test), wild tomato violin plots are shown in blue and cultivated tomato plots are shown in red, otherwise the common pattern is shown in red. Stages of fruit development on the X-axis are numbered sequentially followed by “Br” for breaker stage and “RR” for red ripe stage. Note that panels have independent Y-axis to maximise readability. [file Image_4.jpeg]

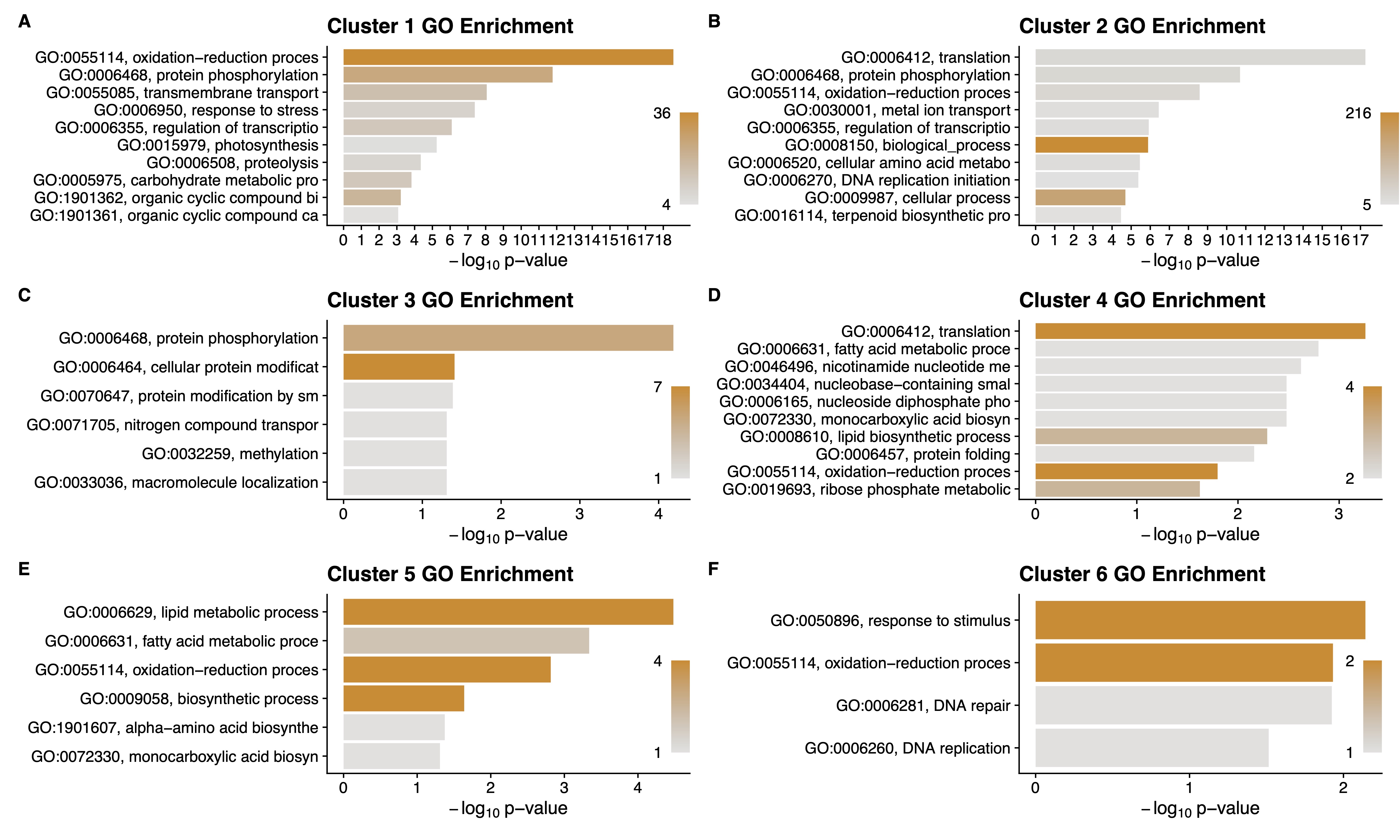

Supplement: Supplementary Figure 5 — GO Enrichment analysis for desert tobacco gene expression clusters in . GO term descriptions to the left of the enrichment graphs are truncated for space and sorted by p-value. The bars are colored by the number of genes assigned to each GO term with legends in the lower right of each graph. Stages of fruit development in the axis of B-GD are numbered sequentially followed by “Tr” for transition to mature stage. [file Image_5.jpeg]

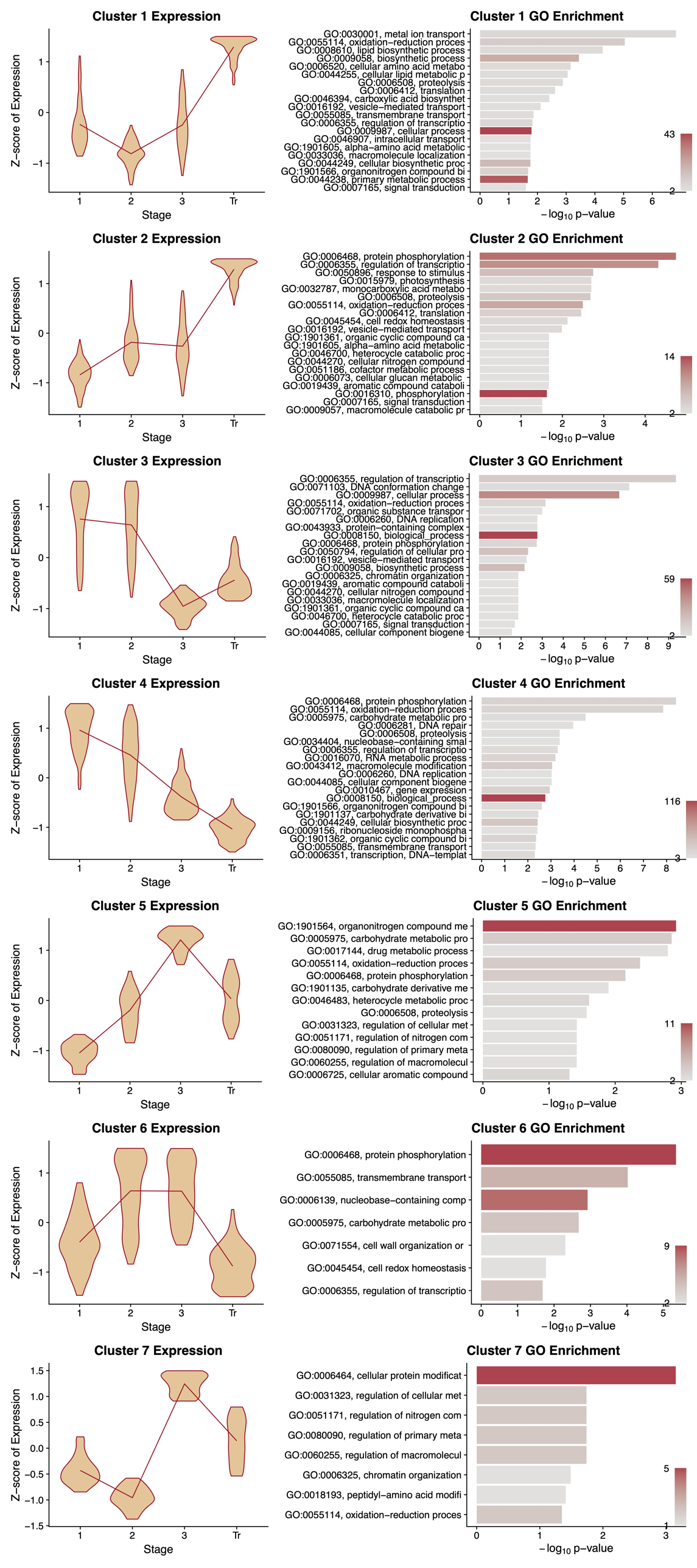

Supplement: Supplementary Figure 6 — Summary of clustered gene expression profiles for genes with conserved patterns among the three solanaceous species. Violin plots of normalised expression by developmental stage for each cluster are shown on the left and gene ontology (GO) enrichment plots for the genes in the corresponding cluster are shown on the right. GO term descriptions to the left of the enrichment graphs are truncated for space and sorted by p-value. The bars are colored by the number of genes assigned to each GO term with legends in the lower right of each graph. Stages of fruit development in the axis are numbered sequentially followed by “Tr” transition to mature stage. [file Image_6.jpeg]

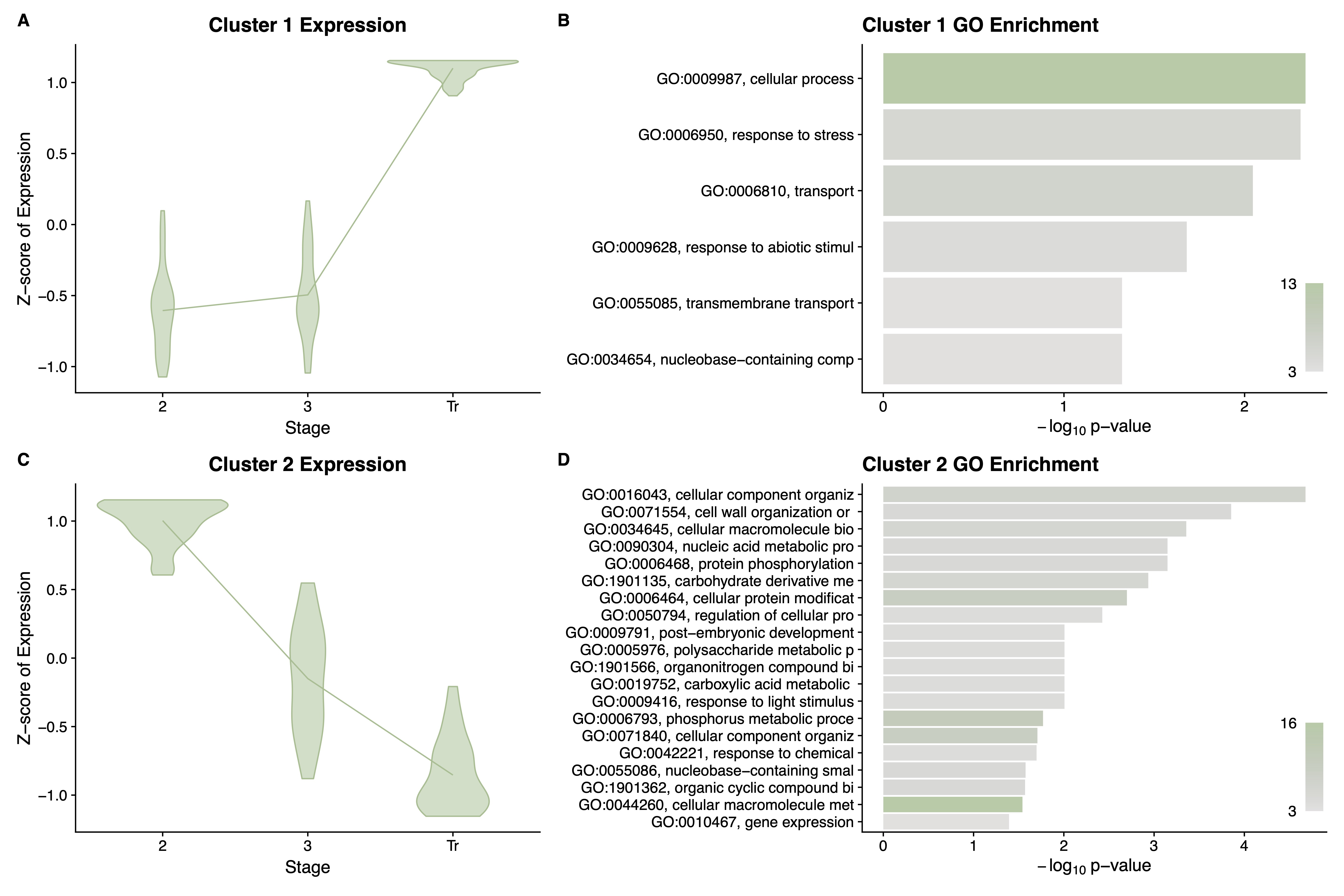

Supplement: Supplementary Figure 8 — Summary of clustered gene expression profiles for genes with conserved patterns among the five species. Violin plots of normalised expression by developmental stage for each cluster are shown on the left and gene ontology (GO) enrichment plots for the genes in the corresponding cluster are shown on the right. GO term descriptions to the left of the enrichment graphs are truncated for space and sorted by p-value. The bars are colored by the number of genes assigned to each GO term with legends in the lower right of each graph. Stages of fruit development in the axis are numbered sequentially followed by “Tr” transition to mature stage. [file Image_8.jpeg]
